# Mobile Molecules: Reactivity Profiling Guides Faster Movement on a Cysteine Track

**DOI:** 10.1002/ange.202300890

**Published:** 2023-04-13

**Authors:** Zonghua Bo, Zhong Hui Lim, Fernanda Duarte, Hagan Bayley, Yujia Qing

**Affiliations:** ^1^ Department of Chemistry University of Oxford Mansfield Road Oxford OX1 3TA UK

**Keywords:** Mobile Molecules, Nanopores, Protein Engineering, Single-Molecule Chemistry, Thiol-Disulfide Interchange

## Abstract

We previously reported a molecular hopper, which makes sub‐nanometer steps by thiol‐disulfide interchange along a track with cysteine footholds within a protein nanopore. Here we optimize the hopping rate (ca. 0.1 s^−1^ in the previous work) with a view towards rapid enzymeless biopolymer characterization during translocation within nanopores. We first took a single‐molecule approach to obtain the reactivity profiles of individual footholds. The p*K*_a_ values of cysteine thiols within a pore ranged from 9.17 to 9.85, and the pH‐independent rate constants of the thiolates with a small‐molecule disulfide varied by up to 20‐fold. Through site‐specific mutagenesis and a pH increase from 8.5 to 9.5, the overall hopping rate of a DNA cargo along a five‐cysteine track was accelerated 4‐fold, and the rate‐limiting step 21‐fold.

## Graphical abstract

Chemical stepping of biopolymers within a protein nanopore suggests an enzymeless means of sequence characterization. Using a nanoreactor approach, we elucidated the reactivity profile of cysteine footholds along a protein track. Faster chemical stepping was achieved by rational mutagenesis and optimization of reaction conditions. In the future, the system has the potential for directional molecular motion at milliseconds per step.

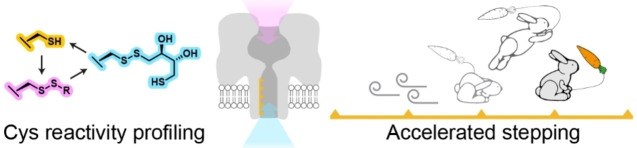

## Introduction

**Figure 1:**
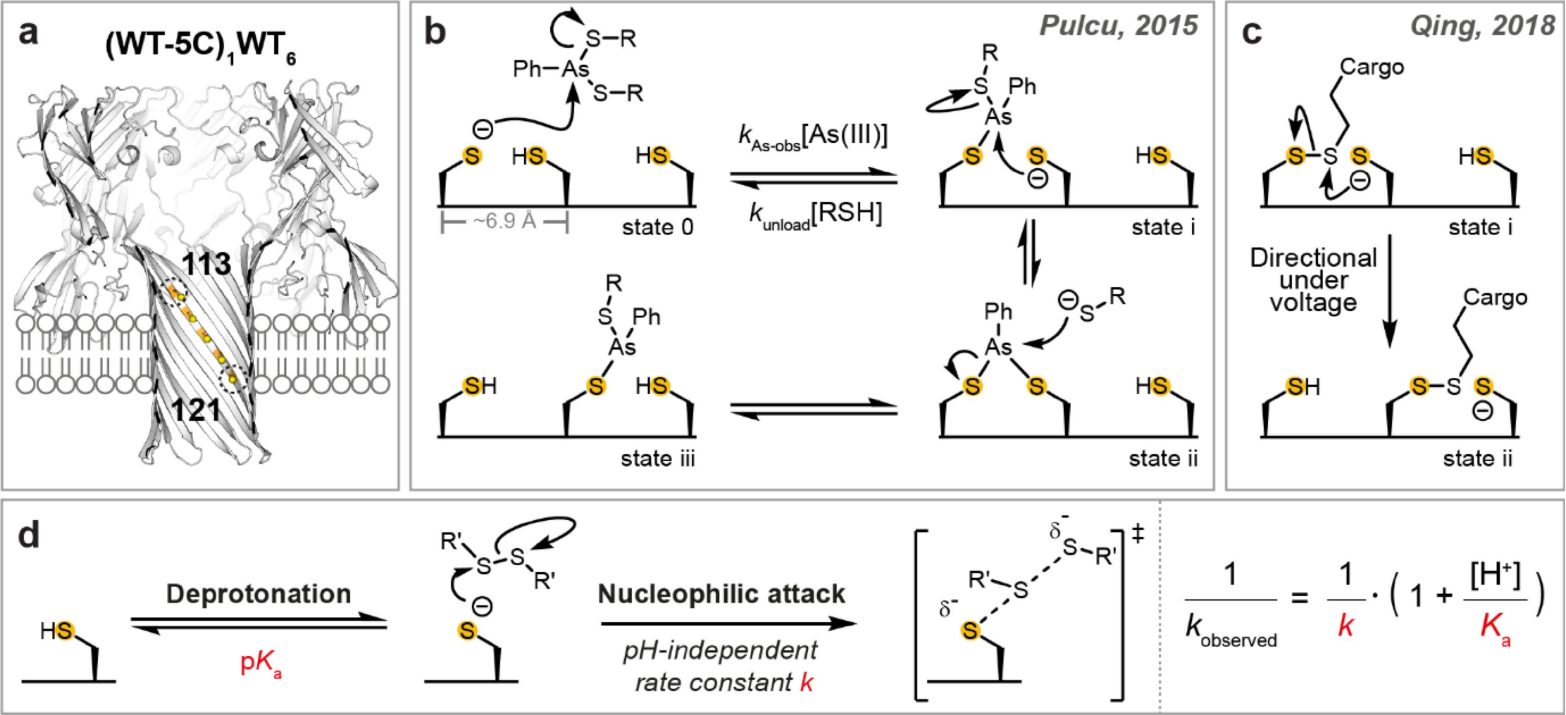
Cargo translocation along a protein track. a) An αHL nanoreactor containing a five‐cysteine track bounded by Cys‐113 and Cys‐121. b) The molecular walker, formed in situ by the reaction of 4‐sulphophenylarsonous acid with excess 2‐(2‐methoxyethoxy)ethanethiol, is referred to as As(III). The walker was first loaded onto the protein track (state 0→i); translocation of the walker (state i→iii) was mediated by two consecutive nucleophilic substitutions.^[8]^ c) Chemical stepping of the molecular hopper along the protein track proceeded through thiol‐disulfide interchange (state i→ii). The directionality of translocation was controlled by an externally applied electric potential.^[9]^ d) Reactivity in a thiol‐disulfide interchange reaction can be separated into two components: thiol deprotonation and subsequent nucleophilic attack. The reaction parameters of individual steps were obtained by using the equation shown.

In cells, motor proteins, such as kinesins, dyneins, and myosins, move along microtubule and actin tracks.^[1]^ In contrast to the prevalence of protein‐based tracks in nature, synthetic mobile molecules tread mostly on DNA[^2^, ^3^, ^4^] and small‐molecule tracks,[^5^, ^6^, ^7^] which are often designed for ease of synthesis. Recently, we constructed small‐molecule walkers and hoppers that move along a protein track comprising cysteine footholds positioned approximately 6.9 Å apart along a β strand within an α‐hemolysin (αHL) pore (Figure 1a).[^8^, ^9^] Cysteine is a desirable building block for a protein track, because the side‐chain thiol, when deprotonated, is a powerful nucleophile. Thiolates can participate in various rapid and often reversible chemistries (e.g., thiol‐disulfide interchange and arsenic(III)‐thiol chemistry) that can be used to produce stepwise molecular motion.[^10^, ^11^] We first reported the movement of a two‐legged molecular walker along a five‐cysteine track within the αHL pore (footholds at positions 113, 115, 117, 119 and 121) based on the substitution reactions of arsenic(III) compounds with thiols (Figure 1b).^[8]^ Later, we developed a single‐legged molecular hopper that carries a DNA cargo and moves along the cysteine track using thiol‐disulfide interchange (Figure 1c).^[9]^ Under an electric potential, the hopping motion was autonomous, processive, and directional. The molecular hopper moves at a stepping rate averaging 0.13 s^−1^ at pH 8.5. The rate varies by up to an order of magnitude depending on the position on the track.^[9]^ Later, the molecular hopper was elaborated into an enzymeless system to effect directional stepwise translocation of biopolymers for sequence analysis.^[12]^ Future practical use of the enzymeless system will require both a longer track and more rapid stepping. To address the latter, we assessed the reactivity of individual cysteine footholds comprising the molecular track to determine how the kinetics of mobile molecules on cysteine tracks can be manipulated.

**Figure 2:**
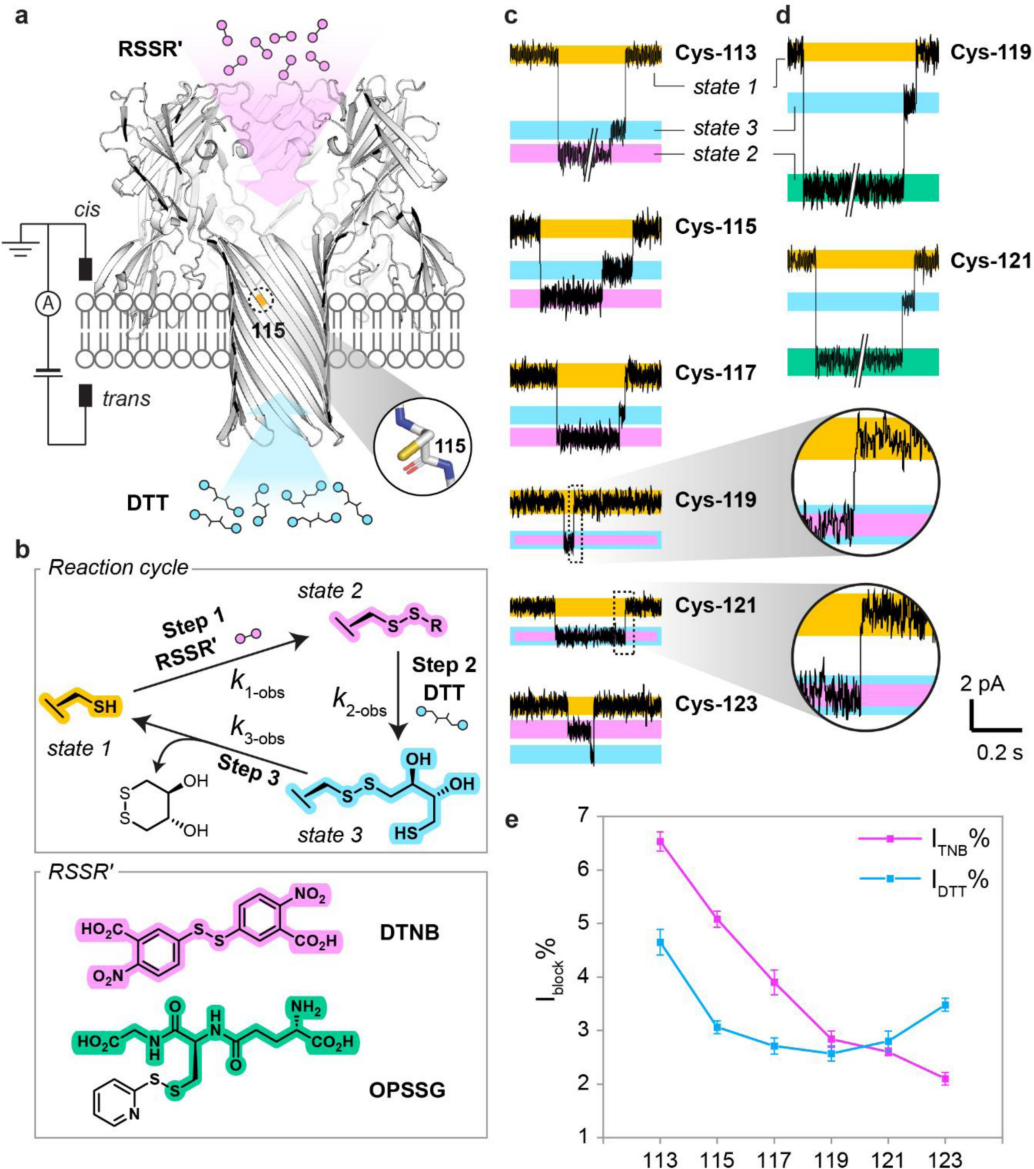
Single‐molecule approach to the determination of p*K*_a_ values for cysteine residues. a) Reactants RSSR′ and DTT, spatially separated by the lipid bilayer, were introduced from the *cis* and *trans* compartments respectively. Cys‐115 is shown as an example of a residue with a reactive side chain. b) The thiol‐disulfide interchange reaction cycle used for p*K*_a_ determination. In each experiment, either DTNB or OPSSG was introduced into the *cis* compartment. c) Single‐channel current recordings depicting complete reaction cycles with DTNB for each single‐cysteine nanoreactor. d) Reaction cycles for OPSSG. The levels in C and D were color‐coded: free cysteine side chain (yellow), αHL‐TNB (pink), αHL‐SG (green) and αHL‐DTT (blue). For Cys‐119 and Cys‐121, the αHL‐DTT level could be discriminated from the αHL‐SG level, but not from the αHL‐TNB level (pink with blue outline). e) Percentage current blockades (I_block_%) for αHL‐TNB and αHL‐DTT for positions 113 to 123.

We first took a nanoreactor approach to probe the reactivity of individual cysteine footholds at the single‐molecule level.^[13]^ Reactivity in the context of the thiol‐disulfide interchange that underlies chemical hopping was determined from the p*K*_a_ values of the cysteine thiols and the pH‐independent rate constants for nucleophilic attack by the corresponding thiolates on a set electrophile (Figure 1d). Single‐cysteine mutations were made at positions 113, 115, 117, 119, 121 or 123. Nanoreactors containing one mutant subunit were prepared, and named (M113C)_1_WT_6_, (T115C)_1_WT_6_, (T117C)_1_WT_6_, (G119C)_1_WT_6_, (N121C)_1_WT_6_, and (N123C)_1_WT_6_. Each nanoreactor was inserted into a planar lipid bilayer for electrical recording. In the presence of small‐molecule disulfide and thiol reagents, spatially separated by the lipid bilayer, thiol‐disulfide interchange reactions occurred at the cysteine site in a cycle of three steps (Figure 2a).^[13]^ In Step 1, a disulfide molecule (RSSR′) entered from the *cis* side to react with the cysteine side chain (state 1) to generate an αHL‐SR′ mixed disulfide (state 2). In Step 2, the mixed disulfide was attacked by DL‐dithiothreitol (DTT) introduced from the *trans* side to generate an αHL‐DTT mixed disulfide (state 3), which then self‐cyclized in Step 3 (Figure 2b). These thiol‐disulfide interchange reactions were monitored by changes in the ionic current passing through the αHL nanoreactor under an applied transmembrane electric potential; the current reflected the bond‐making and bond‐breaking events in real time (Figure 2c). Rate constants were obtained at different pH values from the mean lifetimes of each current state^[14]^ and used to calculate the p*K*_a_ values of the cysteine thiols and the pH‐independent rate constants of the thiolates for attack on the disulfide.

In addition, we modified the environment in the vicinity of Cys‐113 on the molecular track by mutating position 111 to alter the rate of thiol‐disulfide interchange at this position. A mutation that increased the rate was found, which was later incorporated into protein tracks. Finally, chemical stepping of a DNA molecule on a modified cysteine track was tested at two pH values; faster chemical stepping was observed at a higher pH value.

## Results and Discussion

### Single‐Molecule Reactivity Profiling of Individual Cysteine Residues

To monitor the three‐step reaction cycle repeatedly at individual cysteine sites (i.e., Cys‐113, Cys‐115, Cys‐117, Cys‐119, Cys‐121 and Cys‐123) we first treated individual cysteine nanoreactors with 5,5′‐dithiobis(2‐nitrobenzoic acid) (DTNB) and DTT (Figure 2b). Current blockades of the mixed disulfides (I_TNB_% or I_DTT_%) were calculated as a percentage of the open pore current (state 1). We found that the αHL‐TNB and αHL‐DTT adducts formed at positions 119 and 121 were indistinguishable in terms of current blockade (I_TNB_%=I_DTT_%, Figure 2c). I_TNB_%>I_DTT_% was found at positions 113, 115, and 117, whereas I_TNB_%<I_DTT_% was observed at position 123 (Figure 2e). Spatial blockades of the αHL‐TNB or αHL‐DTT adducts at Cys‐113, Cys‐117, and Cys‐121, defined as the end‐to‐end distance of the adduct divided by the radius of the unreacted pore at the cysteine position, were calculated over 600 ns of cumulative molecular dynamics simulations. We observed a linear correlation between the relative simulated spatial blockades and the relative experimental current blockades (see the Supporting Information Section 1 for supplementary discussion).

To resolve the current levels of αHL‐SR′ and αHL‐DTT at Cys‐119 or Cys‐121, we treated (G119C)_1_WT_6_ or (N121C)_1_WT_6_ with orthopyridyl glutathionyl disulfide (OPSSG) and DTT. The αHL‐SG adduct blocked significantly more current than the αHL‐DTT adduct, allowing current level resolution (Figure 2d). However, undesirable regioselectivity was observed in Step 2. αHL‐SG often reacted with DTT to regenerate the nanoreactor cysteine directly without forming an αHL‐DTT adduct, while reaction of αHL‐TNB with DTT exclusively formed αHL‐DTT (see Supporting Information Section 2 for supplementary discussion).

Similar to the foothold thiolates involved in chemical stepping,^[9]^ a nanoreactor cysteine thiolate acted both as the nucleophile (Step 1) and the leaving group (Step 3) during a reaction cycle. To estimate the reactivity of each cysteine thiolate, reaction cycles were recorded using DTNB and DTT at a minimum of three pH values between pH 8.0 and 9.5 using either HEPBS or AMPSO as the buffering agent (see Experimental Details in the Supporting Information). Given that the p*K*_a_ value of a cysteine residue depends on its environment within a protein,^[10]^ an increase in the pH value during a reaction could in principle alter the thiol p*K*_a_ value by 1) deprotonating neighboring ionizable amino acid side chains (if present); 2) eliciting protein conformational changes. We expect that the p*K*_a_ values of individual cysteine thiols are minimally affected within the tested pH range (8.0 to 9.5), because 1) the inward‐facing residues within the β barrel either are non‐ionizable or require a higher pH value for deprotonation (e.g., serine and threonine (p*K*_a_>13)[^15^, ^16^] or lysine (p*K*_a_>10.5)[^17^, ^18^]); 2) the αHL nanopore is stable up to pH 11, with unfolding of the cap domain occurring at pH 12.^[19]^

The observed rate constants for Step 1 or Step 3 (*k*_*i*‐obs_, *i*=1 or 3) were derived from the mean lifetimes of the associated chemical states (⟨*τ_i_* ⟩, *i*=1 or 3). For the bimolecular Step 1, *k*_1‐obs_=1/(⟨*τ*_1_⟩[DTNB]_*cis*_), while for the unimolecular Step 3, *k*_3‐obs_=1/⟨*τ*_3_⟩. To ensure there was no buffer catalysis, we recorded the reaction cycles in (T115C)_1_WT_6_ at pH 8.8, buffered by both HEPBS and AMPSO, and observed no significant difference in *k*_3‐obs_ obtained with the two buffer systems (see Supporting Information Section 3 for supplementary discussion).

Using the Henderson–Hasselbalch equation [Eq. 1a], the pH‐independent rate constants of the thiolate (*k_i_*, *i*=1 or 3) and the p*K*_a_ values of the respective thiols for each nanoreactor were determined by fitting 1/*k*_*i*‐obs_ on [H^+^] using linear regression [Eq. 1b].^[20]^

(1a)

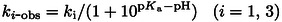



(1b)

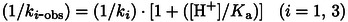




For each nucleophilic thiol, we performed linear regression fitting of 1/*k*_*i*‐obs_ on [H^+^] with 3‐fold cross‐validation to calculate the *k_i_* and the p*K*_a_ values (see Supporting Information Section 4 for supplementary discussion). The p*K*_a_ values of the six cysteine residues ranged from 9.17 (Cys‐113) to 9.85 (Cys‐123; Table 1). The pH‐independent rate constants of the cysteine thiolates for the reaction with DTNB (*k*_1_; Cys‐113, Cys‐115, Cys‐117, Cys‐119, Cys‐121, or Cys‐123) ranged from 2.76±0.21×10^3^ M^−1^ s^−1^ to 5.89±0.52×10^4^ M^−1^ s^−1^, whereas those for intramolecular cyclization of the αHL‐DTT adducts (*k*_3_; Cys‐113, Cys‐115, Cys‐117 or Cys‐123) ranged from 174±38 s^−1^ to 335±48 s^−1^. The errors of the cysteine p*K*_a_ values were less than 0.1, suggesting good data reproducibility despite various sources of experimental error (e.g., determinations of pH, concentration, and temperature) and pore‐to‐pore variation. In comparison to the ensemble pH‐independent rate constants measured for glutathione (Table 1) with DTNB, our *k*_1_ values obtained from single‐molecule experiments at Cys‐115, Cys‐117, Cys‐119 and Cys‐121 were similar (ca. 10^4^ M^−1^ s^−1^), while the *k*_1_ values at Cys‐113 and Cys‐123 were around 10‐fold lower.

**Table 1:**
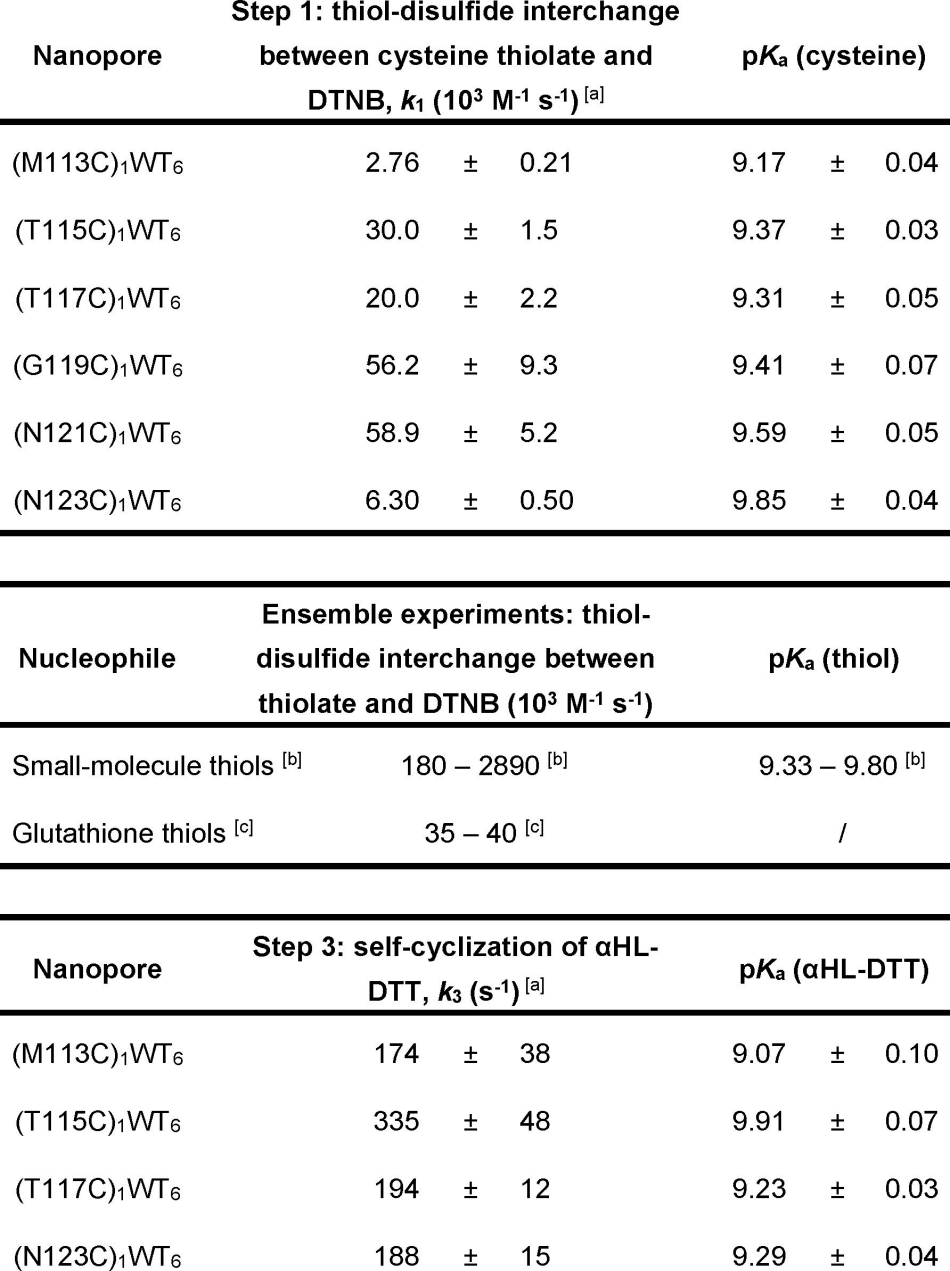
pH‐independent rate constants and p*K*_a_ values of nucleophilic thiols in thiol‐disulfide interchange reactions in single‐molecule experiments and ensemble experiments.

[a] Thiol‐disulfide interchange reactions involved in Step 1 and Step 3 within each nanoreactor were conducted over a range of pH values between 8.0 and 9.5 in 2 M KCl, 50 mM HEPBS/AMPSO adjusted to the appropriate pH value, 20 μM EDTA at 20±1 °C. The applied potential was +50 mV. The p*K*_a_ value of each nucleophilic thiol and the pH‐independent rate constant of the thiolate *k_i_* were calculated from the mean gradient and mean y‐intercept obtained from linear regression fitting with 3‐fold cross‐validation of input values of [H^+^] against the target values of 1/*k*_*i*‐obs_. Standard deviations were calculated using error propagation from standard deviations associated with the mean gradient and mean y‐intercept. [b] Ensemble pH‐independent rate constants and p*K*_a_ values from the literature[^21^, ^22^] for thiols: 2‐mercaptoethanol, 1‐thioglycerol, thioglycolic acid, and methyl 3‐mercaptopropionate. [c] Ensemble pH‐independent rate constants from the literature for glutathione.^[23]^

Our results suggest that the nucleophilicity of cysteine residues in single‐cysteine nanoreactors are not linearly correlated with their p*K*_a_ values. Previous studies of thiol‐disulfide interchange reactions between small‐molecule thiols and disulfides revealed a Brønsted relation wherein the pH‐independent rate constant of the thiolate correlated positively with the p*K*_a_ value of the nucleophilic thiol but negatively with the p*K*_a_ value of the leaving‐group thiol.^[20]^ From these studies, Brønsted coefficients of the nucleophilic thiolate were determined to be 0.4–0.5. In a protein environment, the expected Brønsted relation may be complicated by factors such as electrostatics, sterics, and solvation.[^10^, ^24^, ^25^, ^26^] For example, a study of reactions between disulfides and cysteine thiols embedded in peptides revealed the importance of the net charge of disulfide containing molecules; Brønsted coefficients for the nucleophilic peptide thiolates were 0 and 0.8 for the negatively charged glutathione disulfide and the positively charged cystamine, respectively.^[27]^ Given the small dataset and the narrow p*K*_a_ range covered in the present study, it is unrealistic to extrapolate a free‐energy relationship. Nevertheless, we noted that the poorest nucleophiles corresponded to the cysteine residues with the lowest p*K*_a_ (Cys‐113) and, surprisingly, the highest p*K*_a_ (Cys‐123), with *k*_1_ values approximately an order of magnitude lower than for the remaining cysteine residues.

Previously, an arsenic(III)‐based molecular walker was loaded onto a cysteine track by a thiol‐arsenic interchange reaction between an αHL cysteine and a thiol‐arsenic(III) adduct (Figure 1b).^[8]^ The observed rate constants of walker loading (*k*_As‐obs_) at Cys‐113, Cys‐115, Cys‐117, Cys‐119, and Cys‐121 were measured individually at pH 8.0, and exhibited a similar trend to that of *k*_1‐obs_ (i.e., the observed rate constant for the reaction of a nanoreactor cysteine thiol with DTNB) at pH 8.0, with Cys‐113 being the most slow‐reacting (see Supporting Information Section 5 for supplementary discussion). Thiol‐arsenic exchange reactions were shown to occur with stereochemical inversion,^[28]^ suggesting a collinear transition state (S−As−S) at the arsenic center. This parallels the collinear transition state required for thiol‐disulfide interchange.^[29]^ Hence, the factors influencing the trends observed for the rate constants of thiol‐disulfide interchange (e.g., the p*K*_a_ values of the cysteine thiols, the sterics of the protein environment) might have a parallel effect on thiol‐arsenic exchange.

### Enhanced Cysteine Reactivity by Mutating a Neighboring Residue

**Figure 3:**
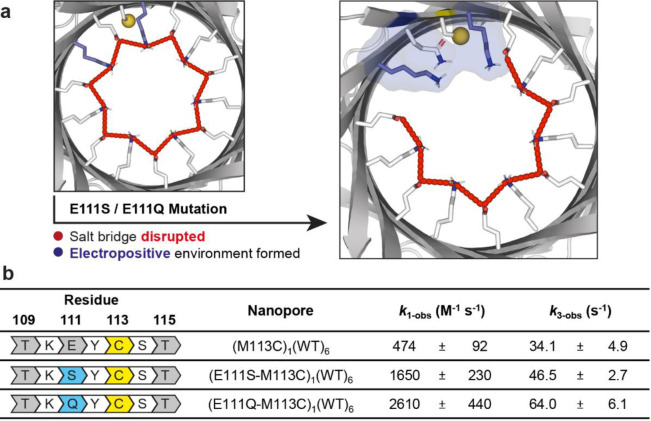
Rational redesign of an engineered track. a) Mutations E111S and E111Q are predicted to disrupt the ring of salt bridges (red dashed lines) and liberate lysine residues (colored in blue). b) Observed rate constants for nucleophilic attack by Cys‐113 on DTNB (*k*_1‐obs_) and intramolecular cyclization of the DTT adducts (*k*_3‐obs_). Dwell‐time analysis and rate constant estimations were performed by using the maximum interval likelihood algorithm of QuB software.^[30]^ Conditions: 2 M KCl, 50 mM HEPBS, pH 8.5, 20 μM EDTA, 20±1 °C.

Noting that the Cys‐113 thiolate had the lowest *k*_1_ value, we engineered the surrounding protein environment to investigate the extent to which its reactivity could be altered. We generated two mutant single‐cysteine nanoreactors, (E111S‐M113C)_1_WT_6_ and (E111Q‐M113C)_1_WT_6_, containing Cys‐113 and either a E111S or E111Q mutation in one of the seven subunits. A net positive charge from Lys‐147 is obtained when the negatively charged Glu‐111 is replaced with the neutral Ser‐111 or Gln‐111. Consequently, the Lys‐147 residues (on the mutant subunit and adjacent wild‐type subunit) are no longer salt‐bridged to the absent Glu‐111 residue (Figure 3a).

Both (E111S‐M113C)_1_WT_6_ and (E111Q‐M113C)_1_WT_6_ gave a stable steady‐state current without frequent gating under ±150 mV, essential for the observation of chemical stepping of a DNA cargo molecule. Both mutations led to an increase in the observed rate constants of thiol‐disulfide interchange (Step 1, *k*_1‐obs_) between Cys‐113 and DTNB by 3.5‐fold (E111S) or 5.5‐fold (E111Q) at pH 8.5 (Figure 3b). Similarly, the observed rate constant of DTT cyclization (Step 3, *k*_3‐obs_) at pH 8.5 increased by 1.4‐fold (E111S) or 1.9‐fold (E111Q; Figure 3b). The electropositive local environment, contributed by the Lys‐147 residues, and hydrogen‐bond donation from Ser‐111 or Gln‐111 residues were hypothesized to stabilize the Cys‐113 thiolate (i.e., lower the p*K*_a_ of Cys‐113). The increased probability of thiol deprotonation increases *k*_1‐obs_.[^31^, ^32^] The greater rate enhancements observed for the E111Q variant implied that a longer side chain enables more favorable interactions for thiol‐disulfide interchange rather than steric hindrance.

The rate enhancement for DTT cyclization (*k*_3‐obs_) is attributed to: a) a lower p*K*_a_ value of the αHL‐DTT thiol producing increased thiol deprotonation, and b) a lower p*K*_a_ value of the Cys‐113 thiol resulting in the thiolate becoming a better leaving group.^[20]^ The rate enhancement for DTT cyclization was lower than that for the preceding Step 1. We speculate this to be due to the αHL‐DTT adduct extending further into the lumen of the pore, away from the Lys‐147 and Ser‐111 or Gln‐111 residues as compared to the Cys‐113 thiol.^[32]^

### Accelerated Chemical Stepping along Engineered Tracks

**Figure 4:**
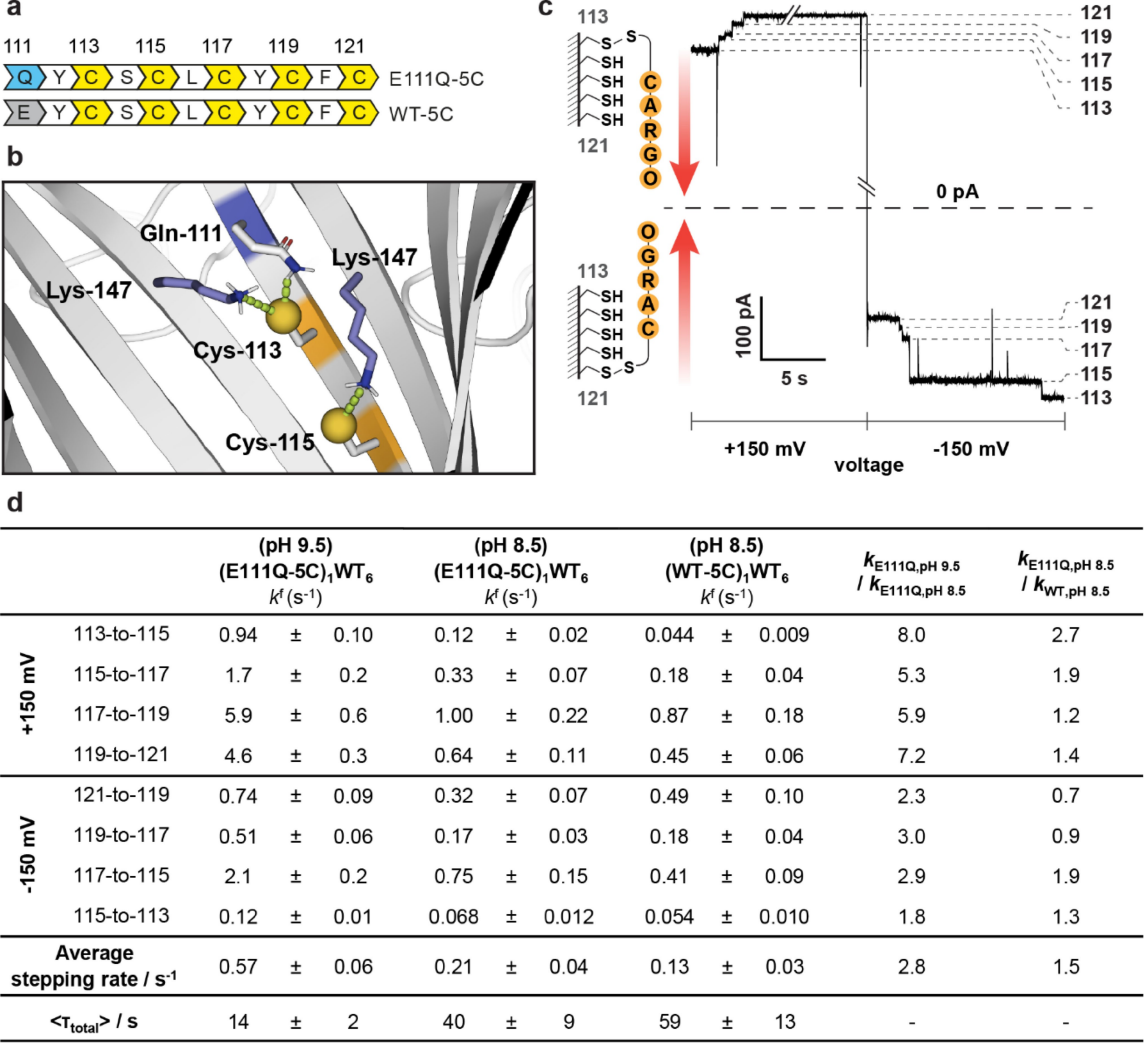
Acceleration of hopping rates along a modified cysteine track. a) Primary sequences of the protein tracks investigated. b) Rate enhancements of steps involving Cys‐115 were attributed to a lowering of the p*K*_a_ value of the Cys‐115 thiol. The Cys‐115 thiolate was stabilized by interaction with the Lys‐147 residue on the adjacent subunit. c) On the five‐cysteine track, four hopping steps were observed at both +150 mV and −150 mV. The direction of the hops is indicated by the red arrows. Conditions: 2 M KCl, 50 mM HEPBS, pH 8.5, 20 μM EDTA, 20±1 °C. d) Hopping rates involving Cys‐113 and Cys‐115 were accelerated under positive (113‐to‐115, 115‐to‐117) and negative potentials (117‐to‐115) on the E111Q‐5C track relative to WT‐5C track. Stepping rates for all steps were accelerated under both positive and negative potentials when the pH value of the buffer was increased to pH 9.5 (50 mM AMPSO). Dwell‐time analysis and rate constant estimations were performed by using the maximum interval likelihood algorithm of QuB software.^[30]^ The average stepping rate along each track was calculated by taking the reciprocal of the average time per step (i.e., average time per step=⟨τ_total_⟩/8).

Encouraged by the ability to increase the reactivity of Cys‐113, we incorporated Gln‐111 into nanoreactors containing a five‐cysteine track. Using PyMOL modeling, we identified favorable hydrogen‐bonding and ionic interactions within the mutant environment to stabilize the deprotonated form of either Cys‐113 (N−S ca. 3.6 Å between Lys‐147 and Cys‐113 or N−S ca. 2.2 Å between Gln‐111 and Cys‐113) or Cys‐115 (N−S ca. 3.3 Å between Lys‐147 and Cys‐115; Figure 4a). These interactions were expected to lower the p*K*_a_ values of both Cys‐113 and Cys‐115 thiols, promoting the formation of a nucleophilic thiolate and improving the leaving group ability of the cysteine thiolate.^[20]^ The (E111Q‐5C)_1_WT_6_ nanoreactor containing the engineered protein track E111Q‐M113C‐T115C‐T117C‐G119C‐N121C was tested with a molecular hopper carrying an oligo‐adenosine 40‐mer cargo (Figure 4a).^[9]^ The results were compared to the (WT‐5C)_1_WT_6_ nanoreactor containing the protein track M113C‐T115C‐T117C‐G119C‐N121C (Figure 4a). On the cysteine track, the hopper moved forward in the direction of Cys‐113 to Cys‐121 under +150 mV, and from Cys‐121 to Cys‐113 under −150 mV. Occasional backstepping could occur when the hopper moved in the reverse direction of that determined by the orientation of the DNA cargo. Both forward and backward steps occur by thiol‐disulfide interchange, where the final foothold acts as the nucleophile, the initial foothold acts as the leaving group, and the hopper sulfur atom acts as an electrophile.

Oligonucleotide translocation in (E111Q‐5C)_1_WT_6_ was first investigated at pH 8.5. We observed faster 113‐to‐115 stepping under +150 mV (*k* ^*f*^_113‐115_=0.12±0.02 s^−1^ in (E111Q‐5C)_1_WT_6_ compared to 0.044±0.009 s^−1^ in (WT‐5C)_1_WT_6_), while 115‐to‐113 stepping under −150 mV remained slow (*k* ^*f*^_115‐113_=0.068 ±0.012 s^−1^ in (E111Q‐5C)_1_WT_6_ compared to 0.054±0.010 s^−1^ in (WT‐5C)_1_WT_6_; Figure 4d). We attribute the variations in rate enhancement to differences in DNA conformational lability under opposite potentials. When the DNA is bonded to Cys‐113 and oriented towards the *trans* opening of the pore at +150 mV, about 4 phosphodiester groups lie within the electric field, which for the most part drops along the length of the β barrel. In contrast, an oligonucleotide bonded to Cys‐115 and oriented towards to the *cis* opening of the pore at −150 mV would only have about 1 phosphodiester group within the electric field.^[9]^ Therefore, the stronger electrophoretic force acting on the DNA under +150 mV might align the disulfide bond linking the oligonucleotide to Cys‐113 with Cys‐115; by contrast, the oligonucleotide bonded to Cys‐115 might have more degrees of freedom under −150 mV, which reduces occupation of the collinear conformation required for stepping to Cys‐113.^[29]^ Previous computational studies have shown that deviation of the collinear transition state from a S−S−S angle of 172° to 108° leads to an increase in activation energy of 167 kJ mol^−1^.^[33]^ As predicted by PyMOL modeling, we also recorded an approximately 2‐fold increase in the rate for the 115‐to‐117 step under +150 mV and for the 117‐to‐115 step under −150 mV in (E111Q‐5C)_1_WT_6_ (Figure 4d). The former is attributed to the Cys‐115 thiolate being a better leaving group, and the latter to increased Cys‐115 thiol deprotonation. All other steps occurred at similar rates by comparison to those in (WT‐5C)_1_WT_6_.

We next studied oligonucleotide translocation at pH 9.5 in (E111Q‐5C)_1_WT_6_. The rates of individual hopping steps at pH 9.5 increased by 5‐ to 8‐fold under +150 mV and 2‐ to 3‐fold under −150 mV by comparison with those measured at pH 8.5. Using the Henderson–Hasselbalch equation with the p*K*_a_ values derived for individual cysteine residues, we predicted a 4‐ to 6‐fold increase in stepping rates based on increased thiol deprotonation, which was in agreement with the observed rates under +150 mV. Smaller rate increases for steps under a potential of −150 mV could again be attributed to the DNA having more degrees of freedom, thereby reducing occupation of the linear conformation required for thiol‐disulfide interchange. While rate enhancement for the 115‐to‐113 step under −150 mV was low, the directionality of translocation was unaffected by 115‐to‐117 backstepping, with the rate of forward stepping (*k* ^*f*^_115‐113_=0.12±0.01) being an order of magnitude greater than that of backstepping (*k* ^*b*^_115‐117_=0.012±0.005) in (E111Q‐5C)_1_WT_6_. Rates for other backsteps were not calculated owing to insufficient numbers of events (i.e., 14 backsteps out of 654 forward steps in (E111Q‐5C)_1_WT_6_ at pH 9.5, 7 of which were 115‐to‐117 backsteps under −150 mV).

For enzymeless sequencing, the biopolymer has to be moved from one end of the track to the other repeatedly for the measurement of current blockades. The mean total transit time (⟨*τ*_total_⟩) is defined as the time taken to complete a full cycle along a given track (i.e., from Cys‐113 to Cys‐121 under +150 mV followed by Cys‐121 to Cys‐113 under −150 mV). At pH 8.5, the E111Q mutation reduced ⟨*τ*_total_⟩ from 59±13 s in (WT‐5C)_1_WT_6_ to 40±9 s in (E111Q‐5C)_1_WT_6_. In particular, the E111Q mutation reduced the mean dwell time of the slowest step at pH 8.5 (i.e., 113‐to‐115 under +150 mV) from 23±5 s to 8.4±1.7 s, accounting for 70 % of the overall transit time reduction. If a uniform track with no hold‐ups were desired, site‐directed mutagenesis provides a means of targeting the slowest steps. A pH change from 8.5 to 9.5 further shortens ⟨*τ*_total_⟩ to 14±2 s in (E111Q‐5C)_1_WT_6_. Rate acceleration of the two slowest steps (i.e., 113‐to‐115 under +150 mV and 115‐to‐113 under −150 mV) accounted for 56 % of the overall transit time reduction. The average stepping rate was 0.13±0.03 s^−1^ in (WT‐5C)_1_WT_6_ at pH 8.5, and 0.21±0.04 s^−1^ and 0.57±0.06 s^−1^ in (E111Q‐5C)_1_WT_6_ at pH 8.5 and pH 9.5, respectively (Figure 4d).

## Conclusion

In conclusion, the mean total transit time for DNA cargo translocation was shortened from 59 to 14 s. Each step in (E111Q‐5C)_1_WT_6_ at pH 9.5 averaged 0.57 s^−1^, a 4‐fold increase in rate from the original system (i.e., (WT‐5C)_1_WT_6_ at pH 8.5). The rate‐limiting step in the (WT‐5C)_1_WT_6_ nanoreactor was accelerated 21‐fold (*k* ^*f*^_113‐115_=0.044±0.009 s^−1^ in (WT‐5C)_1_WT_6_ at pH 8.5 as compared to 0.94±0.10 s^−1^ in (E111Q‐5C)_1_WT_6_ at pH 9.5).

Our results from site‐directed mutagenesis indicate that favorable changes to cysteine reactivity can be elicited by introducing the E111Q mutation. In this study, a 3.5‐ to 5.5‐fold increase in reactivity was observed towards the small‐molecule disulfide DTNB, while a 2‐ to 3‐fold increase in reactivity was observed for selected steps on the cysteine track. All hopping steps within the (E111Q‐5C)_1_WT_6_ nanoreactor were sped up by increasing the pH value from 8.5 to 9.5. Without further track engineering, faster translocation could be achieved at a pH value above 9.5, where all cysteine footholds are fully deprotonated. We observed asymmetric rate enhancements under +150 mV and −150 mV in the presence of the E111Q mutation and the pH increase. We speculate that the DNA cargo adopts different conformations under opposite potentials; the collinear transition state for thiol‐disulfide interchange will be accessed less often when the DNA cargo has more degrees of freedom.^[29]^

The kinetics of mobile molecules might also be influenced by increasing the temperature. Previously, protein pores leukocidin, OmpG and various αHL mutants were shown to be stable in single‐channel recordings when the temperature was brought to more than 90 °C.^[34]^ By this means, the binding of β‐cyclodextrin to the αHL mutant (M113N)_7_ was shown to follow the linear form of the Van't Hoff equation. Using an activation energy of 50 kJ mol^−1^,^[35]^ a 50 °C increase in temperature from 25 to 75 °C is predicted to produce an 18‐fold rate acceleration for thiol‐disulfide interchange. Assuming each step is accelerated 18‐fold, the mean total transit time in (E111Q‐5C)_1_WT_6_ would be reduced to 0.78 s. Alternatively, selenocysteines might be incorporated into the protein track in place of cysteine. Selenolate (i.e., a deprotonated selenol) would act as the nucleophile and the leaving group during each hopping step. If selenium were incorporated into the biopolymer cargo, selenium could act as the electrophile for nucleophilic attack during each hopping step. Previous studies have suggested that 1) selenolates are approximately 1 order of magnitude more reactive than thiolates as nucleophiles; 2) selenium atoms are approximately 4 orders of magnitude more reactive than sulfur atoms as electrophiles.^[36]^ Incorporation of an electrophilic selenium as the cargo handle could greatly reduce the mean total transit time to <1 ms, equivalent to >1000 complete cycles across the track per second.

Our system can be generalized for the translocation of biopolymers other than DNA, such as polypeptides. While the rates in our system do not so far compare to those of the helicase motors used in nanopore DNA/RNA sequencing devices, which make hundreds of steps per second, equivalent enzymes do not exist for other biopolymers, making sequencing by chemical stepping an attractive option. Furthermore, with optimization of conditions (i.e., increased pH value and temperature, the use of selenium, etc.), chemical stepping along cysteine tracks could be improved to provide rates comparable to those seen for sequencing devices for nucleic acids. We believe an ideal track for biopolymer translocation should have the following properties: 1) individual steps should be observable, and each foothold should be differentiated by the ionic current; 2) each track should support repeated translocation; 3) the track should be readily tunable by site‐directed mutagenesis or chemical modification; 4) stepping rates should be similar throughout the track to avoid hold‐ups during translocation. The present study presents significant advances in our endeavor to build an ideal track.

## Conflict of interest

Hagan Bayley is the Founder of, a consultant for and a shareholder of Oxford Nanopore Technologies, a company engaged in the development of nanopore sensing and sequencing technologies.

1

## Supporting information

As a service to our authors and readers, this journal provides supporting information supplied by the authors. Such materials are peer reviewed and may be re‐organized for online delivery, but are not copy‐edited or typeset. Technical support issues arising from supporting information (other than missing files) should be addressed to the authors.

Supporting Information

## Data Availability

The data that support the findings of this study are available from the corresponding authors upon reasonable request.
