# Supplementary material for: Mobile Molecules: Reactivity Profiling Guides Faster Movement on a Cysteine Track
Source: Angew Chem Weinheim Bergstr Ger. 2023 Apr 13;135(21):e202300890. doi: 10.1002/ange.202300890 (PMC10962685; doi:10.1002/ange.202300890)
Supplement: Supplementary file 1 — Supporting Information [file ANGE-135-0-s001.pdf]

Angewandte Chemie
Eine Zeitschrift der Gesellschaft Deutscher Chemiker

Supporting Information

Mobile Molecules: Reactivity Profiling Guides Faster Movement on a Cysteine Track

Z. Bo, Z. H. Lim, F. Duarte, H. Bayley*, Y. Qing**

Table of Contents

List of Abbreviations S3

Section 1. **Computational studies of current blockades by αHL adducts** S4
Section 2. **Regioselectivity of attack in αHL-SG adducts** S10
Section 3. **Buffer catalysis** S11
Section 4. **Linear regression analysis** S12
Section 5. **Observed rate constants for thiol-arsenic exchange** S15
Section 6. **Further analysis of rate constant k_3** S16
Section 7. **Biopolymer translocation in (E111Q-2C)$_1$WT$_6$** S17

Experimental Details S18
General S18
Plasmid preparation S18
Protein preparation S19
In vitro transcription and translation and preparation of αHL nanoreactors S19
Single-channel electrical recordings S20

References S21

List of Abbreviations

**αHL**	α-hemolysin from *Staphylococcus aureus*
**DTNB**	5,5’-dithio-bis-(2-nitrobenzoic acid)
**DTT**	DL-dithiothreitol
**OPSSG**	Orthopyridyl glutathionyl disulfide
**Cys-113**	Cysteine residue at position 113
**WT**	Wild-type subunit of α-hemolysin from *Staphylococcus aureus*
**$(M113C)_1WT_6$**	Single-cysteine nanoreactor containing six wild-type subunits and one mutant subunit with a cysteine mutation at position 113.
**$(T115C)_1WT_6$**	Single-cysteine nanoreactor containing six wild-type subunits and one mutant subunit with a cysteine mutation at position 115.
**$(T117C)_1WT_6$**	Single-cysteine nanoreactor containing six wild-type subunits and one mutant subunit with a cysteine mutation at position 117.
**$(G119C)_1WT_6$**	Single-cysteine nanoreactor containing six wild-type subunits and one mutant subunit with a cysteine mutation at position 119.
**$(N121C)_1WT_6$**	Single-cysteine nanoreactor containing six wild-type subunits and one mutant subunit with a cysteine mutation at position 121.
**$(N123C)_1WT_6$**	Single-cysteine nanoreactor containing six wild-type subunits and one mutant subunit with a cysteine mutation at position 123.
**$(E111S\text{-}M113C)_1WT_6$**	Single-cysteine nanoreactor containing six wild-type subunits and one mutant subunit with a serine mutation at position 111 and a cysteine mutation at position 113.
**$(E111Q\text{-}M113C)_1WT_6$**	Single-cysteine nanoreactor containing six wild-type subunits and one mutant subunit with a glutamine mutation at position 111 and a cysteine mutation at position 113.
**$(WT\text{-}5C)_1WT_6$**	Five-cysteine nanoreactor containing six wild-type subunits and one mutant subunit with five cysteine mutations at positions 113, 115, 117, 119 and 121.
**$(E111Q\text{-}2C)_1WT_6$**	Two-cysteine nanoreactor containing six wild-type subunits and one mutant subunit with a glutamine mutation at position 111 and two cysteine mutations at positions 113 and 115.
**$(E111Q\text{-}5C)_1WT_6$**	Five-cysteine nanoreactor containing six wild-type subunits and one mutant subunit with a glutamine mutation at position 111 and five cysteine mutations at positions 113, 115, 117, 119 and 121.

1. Computational studies of current blockades by αHL adducts

1.1 Model preparation

Because the thiol-disulfide interchange reaction occurs only within the β barrel of the αHL nanopores, a minimal β barrel model was constructed.[1] In this model, residues Glu-111 to Asn-123, which are in or close to the β barrel region were kept, and all residues outside the β barrel region were deleted. To further reduce model complexity, all membrane-facing sidechains were removed. Six systems, αHL-TNB or αHL-DTT adducts at positions 113, 117 or 121, were prepared. Force field parameters for the cysteine residues carrying TNB or DTT molecules were obtained from the GAFF force field.[2,3] For the modified residues, partial charges were determined with the Restrained Electrostatic Potential (RESP) method at the HF/6-31G* level using Gaussian16[4] and ANTECHAMBER.[2,3]

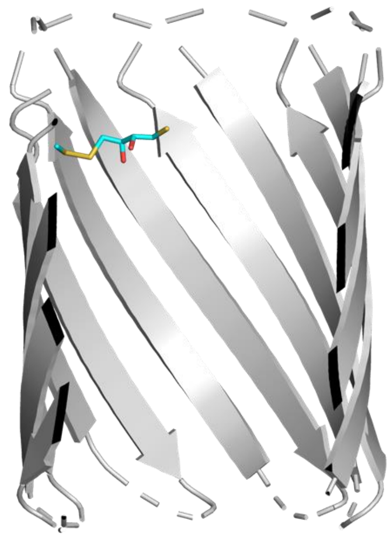

Figure S1. The model pore system setup. A αHL-DTT adduct (cyan) at position 113 in truncated pore is (grey) shown as an example.

1.2 Molecular dynamics simulation protocol

Molecular dynamics (MD) simulations were performed with GROMACS (v2019.2).[5] For each system, the nanopore was modeled with the AMBER99SB-ILDN force field and inserted into a cubic box of TIP3P water molecules.[6,7] The system was neutralized and 0.15 M NaCl was added. The system was energy minimized using the steepest descent algorithm until the maximum force was below 1000 kJ mol^{-1} nm^{-1}. A positional restraint of 1000 kJ mol^{-1} nm^{2} was subsequently applied on the protein backbone atoms. Three independent runs were performed with random initial velocities generated according to the Maxwell–Boltzmann distribution at 298 K. The system was equilibrated under a constant volume and temperature (NVT) ensemble at 298 K (100 ps, 2 fs timestep), followed by equilibration under a constant pressure and temperature (NPT) ensemble at 1 bar and 298 K (100 ps, 2 fs timestep). Production simulations were performed using the NPT ensemble at 1 bar and 298 K (200 ns, 2 fs timestep). An electric field of 0.008 V nm^{-1} (equivalent to ~50 mV across the membrane) was applied in the Z direction across the simulation box. The temperature of the system was maintained at 298 K using the V-rescale thermostat.[8] Pressure was controlled by the Parrinello-Rahman barostat at 1.0 bar, with an isothermal compressibility of 4.5×10^{-5} bar^{-1}.[9] All simulations were performed with three-dimensional periodic boundary conditions. Long-range electrostatics was described with the Particle Mesh Ewald (PME) algorithm.[10,11] All bond lengths involving hydrogen atoms were constrained using the LINCS algorithm.[12] Structures and input files are available on GitHub at https://github.com/duartegroup/Current-blockade-in-nanopore.

1.3 Current blockade and spatial blockade

As shown in Figure 2e in the main text, the percentage current blockade of αHL-TNB (I_{TNB}%) and αHL-DTT (I_{DTT}%) adducts varied at the six positions tested along the β barrel. I_{TNB}% showed a gradual decrease from position 113 to 123, whereas I_{DTT}% exhibited a U-shaped behavior with a minimum at position 119. This was accompanied by a switch in relative magnitude (i.e., $I_{TNB}\% > I_{DTT}\%$ at positions 113-117; $I_{TNB}\% = I_{DTT}\%$ at positions 119 and 121; $I_{TNB}\% < I_{DTT}\%$ at position 123). This shows that current blockade by molecules within an αHL pore is not simply determined by molecular mass.

Spatial blockades of the αHL-TNB or αHL-DTT adducts at Cys-113, Cys-117, and Cys-121, defined as the end-to-end distance of the adduct divided by the radius of the unreacted pores at the cysteine position, were calculated over 600 ns of cumulative MD simulations. The spatial blockade was calculated as the end-to-end distance of an adduct divided by the radius of the unreacted pore at the cysteine positions in the unreacted pores (r_{113} = 5.8 Å, r_{117} = 5.5 Å, r_{121} = 6.4 Å) and labelled as d_{TNB}% and d_{DTT}%. The radius of the unreacted pore at each position was calculated using the HOLE program.[13,14] A positive linear correlation was found between Δd% ($d_{TNB}\% - d_{DTT}\%$) and ΔI% ($I_{TNB}\% - I_{DTT}\%$) (R^2 = 0.98, Figure S2c), suggesting a strong correlation between the relative spatial blockade and the relative current blockade.

The αHL-DTT adducts have multiple conformational states, categorized by the end-to-end distances of the adducts (d_{EE}) (Figure S3). The conformations of the αHL-DTT adducts were predominantly stabilized by hydrogen bonding (HB) interactions between suitably oriented hydroxyl groups of DTT and neighboring residues. For example, at position 113, a HB interaction between a hydroxyl group of DTT and Thr-145 on the adjacent strand (occupancy = 24%) resulted in an extended conformation of αHL-DTT (d_{EE} = 10 Å), whereas a HB interaction with Glu-111 on the same strand (occupancy = 10%) led to a folded conformation (d_{EE} = 4.9 Å). At positions 117 and 121, the αHL-DTT adduct adopted an extended conformation with both hydroxyl groups pointing towards the water-filled channel (d_{EE} = 7.5 Å) with HB interactions with the protein backbone on the adjacent strand: Ser-141 at position 117 (occupancy = 5%), and Gly-122 at position 121 (occupancy = 10%). In comparison, the αHL-TNB adducts had only one major state (Figure S4). This was attributed to the rigidity of the benzene ring, resulting in conformational flexibility mainly coming from the cysteine side chain. A HB interaction was found between the αHL-TNB adduct at position 113 and Thr-145 on the adjacent strand (occupancy = 18%), while no significant HB interactions were found for adducts at positions 117 and 121.

A

Cysteine position	Experimental (I_{block}%)		Computational (d_{EE}%)	
	I_{TNB}%	I_{DTT}%	d_{TNB}%	d_{DTT}%
Cys-113	6.53 ± 0.18	4.65 ± 0.24	15.3 ± 0.1	13.2 ± 0.7
Cys-115	5.08 ± 0.15	3.06 ± 0.12	/	/
Cys-117	3.90 ± 0.23	2.71 ± 0.15	15.3 ± 0.1	13.9 ± 0.3
Cys-119	2.84 ± 0.15	2.57 ± 0.14	/	/
Cys-121	2.60 ± 0.07	2.80 ± 0.19	12.3 ± 0.1	13.4 ± 0.2
Cys-123	2.10 ± 0.12	3.48 ± 0.12	/	/

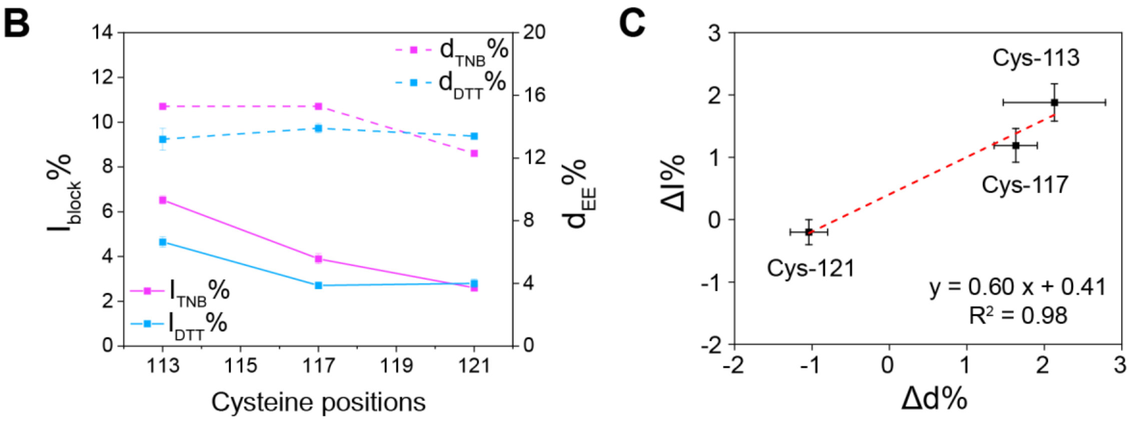

Figure S2. Relationship between current blockade and spatial blockade of the adducts. (A) Summary of percentage current blockades (I_{block}%: I_{TNB}% and I_{DTT}%) and end-to-end distances of the αHL-TNB and αHL-DTT adducts at positions 113, 117 and 121 (d_{EE}%: d_{TNB}% and d_{DTT}%) as a percentage of the radius at the cysteine position of the unreacted pore. For each adduct, the values were calculated over 600 ns of cumulative MD simulations. (B) Comparison between I_{block}% (solid line) and d_{EE}% (dashed line). (C) A linear correlation between Δd% (d_{TNB}% – d_{DTT}%) and ΔI% (I_{TNB}% – I_{DTT}%).

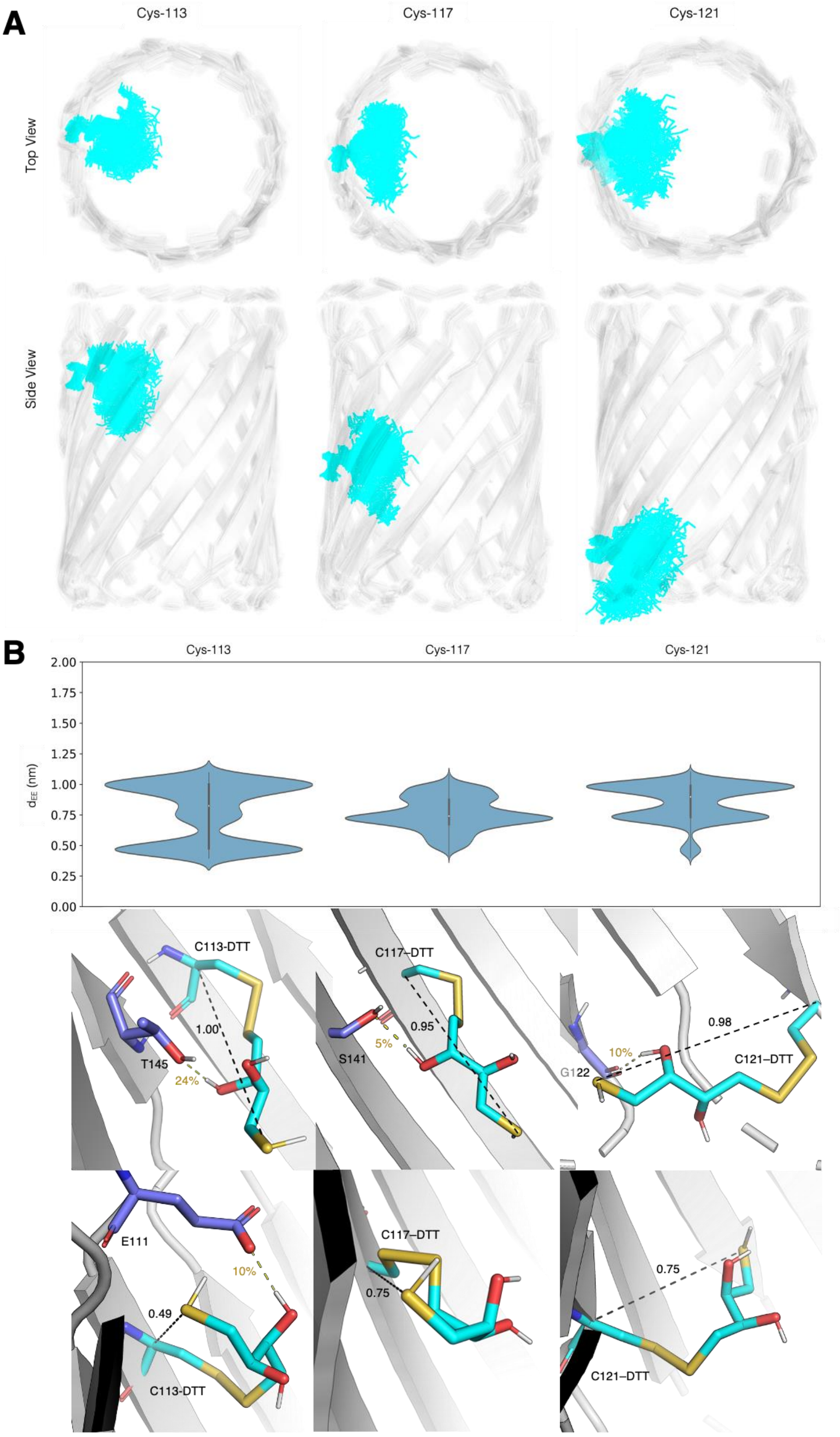

Figure S3. Conformations of the αHL-DTT adducts at positions 113, 117 and 121. (A) Overlap of all conformations of the αHL-DTT adducts (cyan) over 600 ns of cumulative MD simulations at position 113, 117 and 121 respectively. (B) Top: Probability density of the end-to-end distance (d_{EE}) of an αHL-DTT adduct, calculated as the distance between the C_{α} atom of the cysteine residue and the terminal sulfur atom of the αHL-DTT adduct at position 113, 117 and 121 respectively. Bottom: Two most populated conformations of the αHL-DTT adducts (cyan). d_{EE} is indicated with a black dashed line. Hydrogen bonding to a neighboring residue (purple) and its occupancy is indicated in yellow.

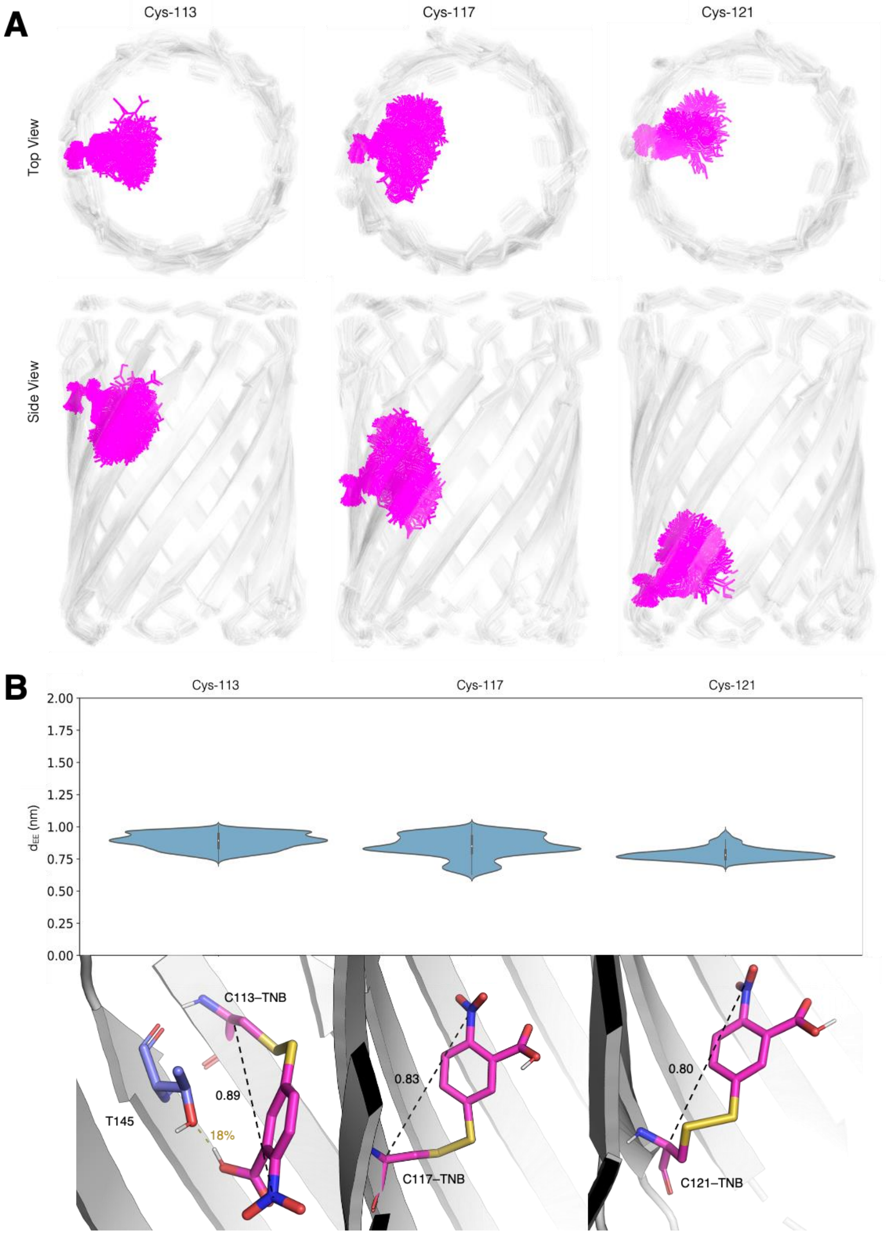

Figure S4. Conformations of the αHL-TNB adducts at positions 113, 117 and 121. (A) Overlap of all conformations of the αHL-TNB adducts (magenta) over 600 ns of cumulative MD simulations at position 113, 117 and 121 respectively. (B) Top: Probability density of the end-to-end distance (d_{EE}) of an αHL-TNB adduct, calculated as the distance between the C_{α} atom of cysteine residue and the terminal nitrogen atom of the αHL-TNB adduct at position 113, 117 and 121 respectively. Bottom: The most populated conformation of the αHL-TNB adducts (magenta). d_{EE} is indicated with a black dash. Hydrogen bonding to a neighboring amino acid (purple) and its occupancy is indicated in yellow.

2. Regioselectivity of attack in αHL-SG adducts

In a mixed disulfide where the two constituent thiols have different acidities, cleavage of the mixed disulfide by a nucleophile would occur preferentially with the release of the more acidic thiol.[15] In Step 2 of the reaction cycle (i.e., thiol-disulfide interchange between DTT and αHL-SR'), DTT could attack either the nanoreactor cysteine thiol (S_{Cys}) or the SR' thiol (S_{SR}). After S_{Cys} is attacked, a short-lived αHL-DTT adduct is formed which subsequently undergoes Step 3 (i.e., intramolecular cyclization to release the nanoreactor cysteine). Alternatively, after S_{SR} is attacked, free cysteine is regenerated directly, bypassing Step 3. We therefore defined regioselectivity as the number of αHL-DTT adducts detected against the overall number of reaction cycles (Table S1). A fraction of higher value indicates that the cysteine thiol S_{Cys} is preferentially attacked over the SR' thiol S_{SR}.

When DTNB was used, Step 2 occurred with complete regioselectivity for S_{Cys} in the αHL-TNB intermediate as thionitrobenzoic acid is a significantly stronger acid (pK_a = 4.5)[16] than the cysteine thiols. When OPSSG was used, Step 1 occurred with complete regioselectivity for the glutathionyl (SG) sulfur to generate the mixed disulfide αHL-SG. This was consistent with the lower pK_a value of OPSH (pK_a = -1.07)[17] compared to that of GSH (pK_a = 8.66).[15] In Step 2 however, regioselectivity for S_{Cys} was diminished as GSH is not a significantly stronger acid than Cys-119 (pK_a = 9.41) or Cys-121 (pK_a = 9.59). Hence, there were reaction cycles where DTT was observed to attack the glutathionyl group, bypassing Step 3 altogether. Regioselectivity for the glutathione adduct αHL-SG at Cys-121 (63%) was five times higher than that at Cys-119 (12%), consistent with the higher *p*K$_a$ value of Cys-121 compared to Cys-119 (Table 1).

Table S1. Regioselectivity of the reaction between DTT and αHL-SG adducts[c]

Cysteine position	Regioselectivity[a] $\frac{n(S_{Cys})}{n(S_{Cys}) + n(S_{SR})}$	$\langle\tau_3\rangle$ (ms)[b]			$k_{3\text{-obs}}$ (s^{-1})[b]		
Cys-119	38/322 (12%)	16	±	3	65	±	10
Cys-121	62/99 (63%)	36	±	5	27	±	4

[a] $n(S_{Cys})$ refers to the number of times the cysteine sulfur was attacked by DTT; $n(S_{SR})$ refers to the number of times the glutathionyl sulfur was attacked by DTT.

[b] τ_3 and $k_{3\text{-obs}}$ refers to the lifetime and rate of intramolecular cyclization of the short-lived αHL-DTT intermediate at pH 8.5 (2 M KCl, 100 mM HEPBS, 20 μM EDTA). OPSSG and DTT were in the *cis* and *trans* compartments respectively.

[c] Dwell time analysis, rate constant estimations and standard error calculations were performed by using the maximum interval likelihood algorithm of QuB.[18]

3. Buffer catalysis

To measure buffer catalysis, the rates of intramolecular cyclization of αHL-DTT (i.e., Step 3) in $(T115C)_1WT_6$ were measured at pH 8.8 with two different buffering agents, HEPBS and AMPSO. The intramolecular cyclization is a unimolecular reaction, the rate of which is less prone to experimental error than the bimolecular Step 1.

A two-tailed test was performed to compare the difference in observed rate constants. We found the *p*-value to be 0.64 (Figure S5). Hence, any observed differences between the two sets of observed rate constants obtained under different buffers is not statistically significant, i.e., no buffer catalysis is observed.

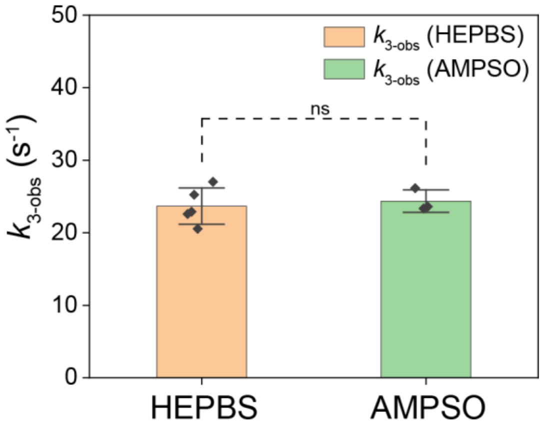

Figure S5. Observed rate constants of intramolecular cyclization of αHL-DTT measured with two buffering agents. Intramolecular cyclization of the αHL-DTT adduct at Cys-115 was investigated at pH 8.8 with either HEPBS or AMPSO. $k_{3\text{-obs}}$ data for Step 3 are plotted; error bars represent the standard deviations of each set of data.

4. Linear regression analysis

In a thiol-disulfide interchange reaction, a thiolate reacts with a disulfide by nucleophilic substitution. The Henderson-Hasselbalch equation gives the ratio of the deprotonated thiolate to the conjugate acid thiol, which is dependent on the pK_a of the thiol and the pH of the reaction medium. Therefore, a linear regression of the observed rate across different pH conditions allows the determination of the pK_a of a thiol and the pH-independent rate constants of the deprotonated thiolate k_i for thiol-disulfide interchange Eq. (1).

$$\frac{1}{k_{\text{i-obs}}} = \frac{1}{k_i} \cdot \left(1 + \frac{[\mathrm{H^+}]}{K_a}\right) \quad (i = 1, 3) \qquad (1)$$

To calculate k_i and pK_a values, we performed linear regression fitting of $1/k_{\text{i-obs}}$ on $[H^+]$ for each thiol. The $[H^+]$ values were calculated from the pH values of the reaction medium. The observed rates for either Step 1 or Step 3 ($k_{\text{i-obs}}$, i = 1 or 3) were derived from the mean lifetime of the corresponding chemical state, $\langle\tau_i\rangle$ (i = 1 or 3). There were ten datasets comprising $[H^+]$ and $1/k_{\text{i-obs}}$ values: six datasets for the cysteine thiol of Cys-113, Cys-115. Cys-117, Cys-119, Cys-121, and Cys-123 involved in Step 1 and four datasets for the free thiols in the αHL-DTT adducts of Cys-113, Cys-115, Cys-117, and Cys-123 involved in Step 3. For each dataset, linear regression analysis with 3-fold cross-validation was performed (Figure S6). Specifically, a dataset was split into 3 groups randomly (labelled A, B and C). To fit Model 1, group C was taken as the test dataset, while the remaining two groups A and B were taken as the training dataset. Similarly, to fit Model 2, group B was taken as the test dataset, while the remaining two groups A and C were taken as the training dataset. To fit Model 3, group A was taken as the test dataset, while the remaining two groups B and C were taken as the training dataset. This means that each datapoint in the dataset was used in a test dataset once and used for linear regression model training twice.

To fit each model, the input values $[H^+]$, denoted as X_{train}, were fitted to the target values $1/k_{\text{i-obs}}$, denoted as y_{train}, using least square linear regression Eq. (2). This yielded a gradient and a y-intercept, denoted as m_j and b_j respectively. To validate the model on the test dataset, predicted target values were calculated using Eq. (3) and then compared to the actual target values. The coefficient of determination ($R^2 > 0.7$) and root mean squared error were used as the accuracy metrics for model evaluation.

$$y_{train} = m_j \cdot X_{train} + b_j \quad (j = 1, 2, 3) \qquad (2)$$

$$y_{pred} = m_j \cdot X_{test} + b_j \quad (j = 1, 2, 3) \qquad (3)$$

In total, three linear regression models were generated for each dataset yielding three gradients (m_1, m_2, and m_3) and three y-intercepts (b_1, b_2, and b_3). The mean gradient and mean y-intercept, denoted as $\bar{m}$ and $\bar{b}$ respectively, were calculated by averaging across the three models (Table S2 and S3). The k_i and pK_a values of the thiols were determined from the mean y-intercept $\bar{b}$ and mean gradient $\bar{m}$ according to Eq. (4) and Eq. (5). Standard deviations in k_i and pK_a were calculated by error propagated from the standard deviations associated with the mean y-intercept $\bar{b}$ and mean gradient $\bar{m}$.

$$k_i = \frac{1}{\bar{b}} \qquad (4)$$

$$pK_a = -\log\left(\frac{\bar{b}}{\bar{m}}\right) \qquad (5)$$

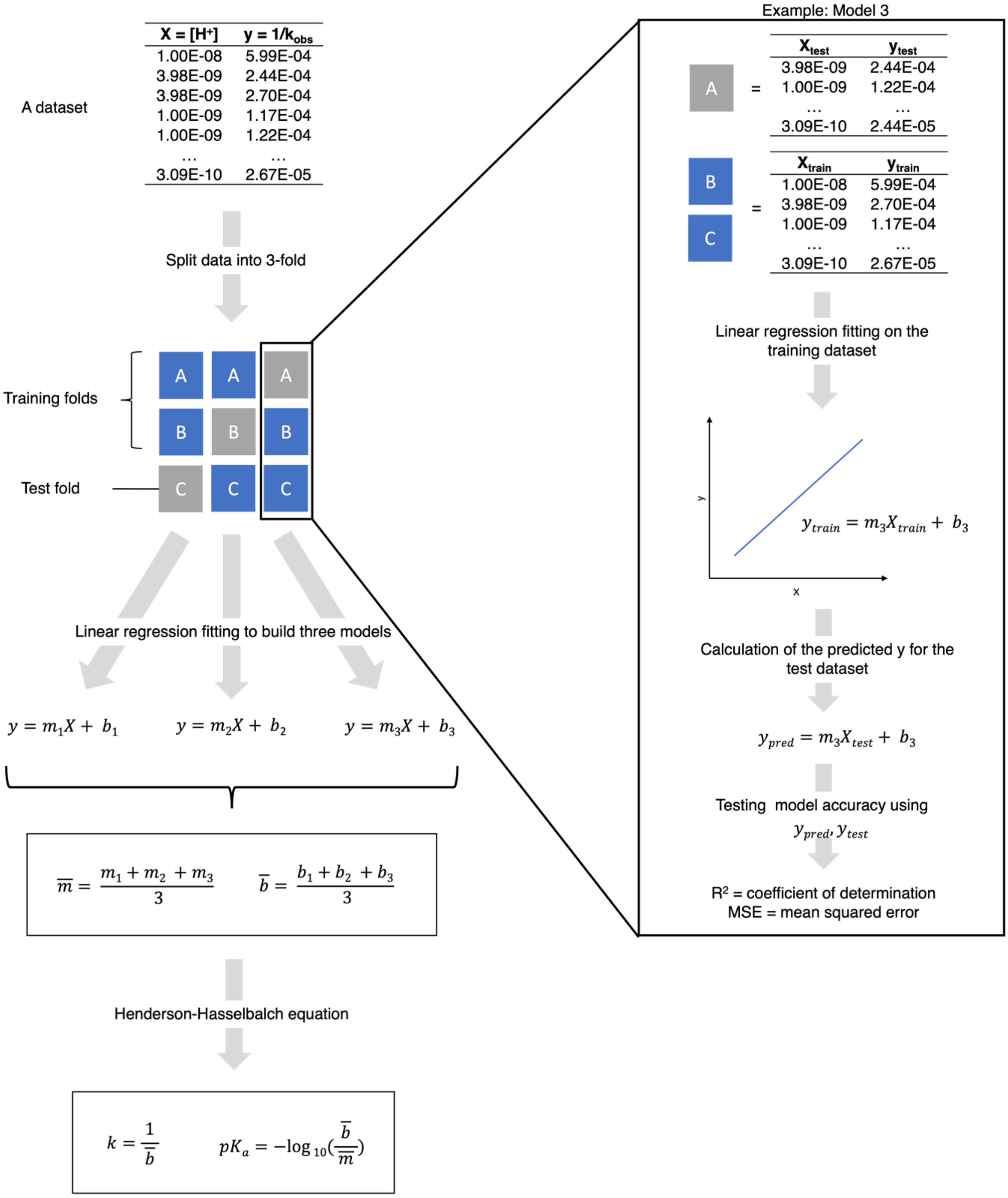

$$\overline{m} = \frac{m_1 + m_2 + m_3}{3} \qquad \overline{b} = \frac{b_1 + b_2 + b_3}{3}$$

$$k = \frac{1}{\overline{b}} \qquad pK_a = -\log_{10}\left(\frac{\overline{b}}{\overline{m}}\right)$$

Figure S6. The workflow of linear regression analysis with 3-fold cross-validation and subsequent calculations using the Henderson-Hasselbalch equation.

Table S2. Summary of gradients, y-intercepts, and accuracy metrics of the linear regression analysis for Step 1.

Cysteine position	Gradient (10^5 M s)	y-intercept (10^{-5} M s)	R^2 score[a]	RMSE (10^{-3})[b]
Cys-113	5.4 ± 0.4	36 ± 3	0.89	0.50
Cys-115	0.78 ± 0.04	3.3 ± 0.2	0.76	0.12
Cys-117	1.0 ± 0.1	4.9 ± 0.4	0.92	0.10
Cys-119	0.45 ± 0.01	1.8 ± 0.1	0.81	0.07
Cys-121	0.66 ± 0.04	1.7 ± 0.2	0.92	0.07
Cys-123	11 ± 1	16 ± 1	0.84	1.8

[a] R^2 score refers to the coefficient of determination of the prediction.

[b] RMSE refers to the root mean squared error between the prediction and the $1/k_{1\text{-obs}}$ in the test dataset.

Table S3. Summary of gradients, y-intercepts, and accuracy metrics of the linear regression analysis for Step 3.

Cysteine position	Gradient (10^5 s)	y-intercept (10^{-5} s)	R^2 score[a]	RMSE (10^{-3})[b]
Cys-113	66 ± 6	602 ± 14	0.85	7
Cys-115	243 ± 14	298 ± 42	0.92	20
Cys-117	87 ± 3	516 ± 32	0.89	10
Cys-123	103 ± 3	518 ± 3	0.89	10

[a] R^2 score refers to the coefficient of determination of the prediction.

[b] RMSE refers to the root mean squared error between the prediction and the $1/k_{3\text{-obs}}$ in the test dataset.

5. Observed rate constants for thiol-arsenic exchange

Single-cysteine αHL mutants were made at positions 113, 115, 117, 119 or 121 and nanoreactors containing one mutant subunit were prepared ($(M113C)_1WT_6$, $(T115C)_1WT_6$, $(T117C)_1WT_6$ etc.). The mutants were reacted with SPAA-MEET$_2$ which was formed *in-situ* from SPAA (*trans* compartment) and MEET (*cis* compartment) (Figure S7a).[19] The observed reaction rates of SPAA-MEET$_2$ with the single-cysteine mutants were directly proportional to the concentration of SPAA-MEET$_2$ (taken to be equal to the concentration of SPAA used) (Figure S7b). The observed rate constant ($k_{As\text{-}obs}$) of the Cys-113 foothold was an order of magnitude lower than the remaining footholds (Figure S7c). A plot of the observed rate constants between the nanoreactors and SPAA-MEET$_2$ at pH 8.0 ($k_{As\text{-}obs}$) showed a similar trend to the observed rate constants for the reaction with DTNB at pH 8.0 ($k_{1\text{-}obs}$) (Figure S7d).

A
B

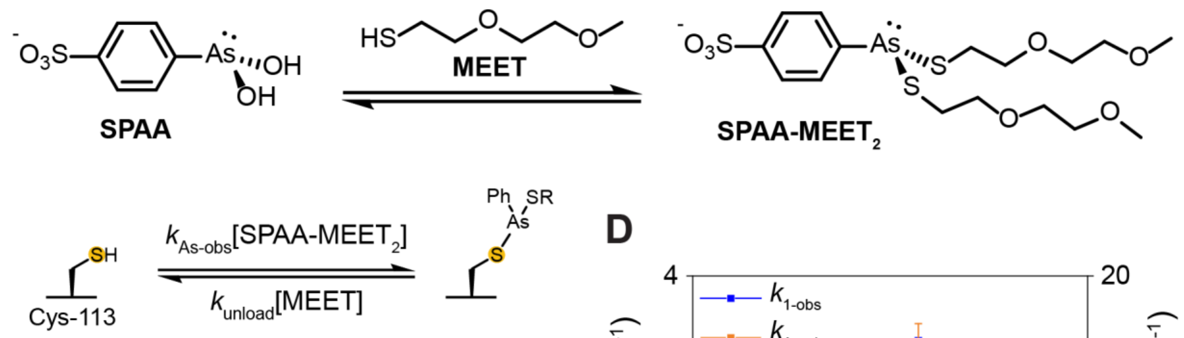

C

System	$k_{As\text{-}obs}$ (10^4 M^{-1} s^{-1})
$(M113C)_1WT_6$	0.3 ± 0.1
$(T115C)_1WT_6$	9 ± 1
$(T117C)_1WT_6$	2.0 ± 0.7
$(G119C)_1WT_6$	15 ± 2
$(N121C)_1WT_6$	5 ± 1

D

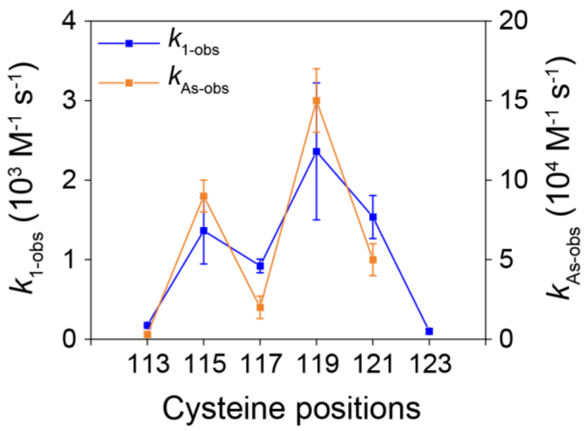

Figure S7. Observed rate constants for reactions between single-cysteine mutants and SPAA-MEET$_2$. (A) Structure of SPAA-MEET$_2$ formed *in-situ*. (B) The observed rate of thiol-arsenic exchange between a single-cysteine mutant (Cys-113 in $(M113C)_1WT_6$ shown here) and SPAA-MEET$_2$ obeys second-order association rates. The reverse reaction obeys second-order association rates and is directly dependent on the concentration of MEET. Experiments for the arsenic walker were performed at pH 8.0 (10 mM bis-tris propane, 2 M KCl, 100 µM EDTA). (C) Observed rate constants ($k_{As\text{-}obs}$) for single-cysteine mutants are tabulated. (D) The observed rate constants for attack on DTNB ($k_{1\text{-}obs}$, blue) followed a similar trend to that for attack on SPAA-MEET$_2$ ($k_{As\text{-}obs}$, orange). Observed rate constants for thiol-disulfide interchange were from experiments conducted at pH 8.0 (2 M KCl, 50 mM HEPBS, 20 µM EDTA).

6. Further analysis of rate constant k_3

The observed rate constants of DTT cyclization were examined at Cys-113, Cys-115, Cys-117 and Cys-123 for a minimum of three pH values between pH 8.0 and 9.5. For Cys-119 and Cys-121, the αHL-DTT levels were indistinguishable from the αHL-TNB levels and could only be distinguished from the αHL-SG levels. The observed rate constants of DTT cyclization for nanoreactors Cys-119 and Cys-121 were only examined at pH 8.5.

The pK_a values for the thiolates in the αHL-DTT adducts were in the range of 9.0 to 10.0 (Table 1), close to the first pK_a value of a DTT molecule (pK_a ~ 9.2).[15] Under the assumption that the rate of initial intermolecular thiol-disulfide interchange was lower than the subsequent intramolecular reaction which releases GSH, the pH-independent bimolecular rate constant for the reduction of GSSG by DTT was calculated to be 37 M^{-1} s^{-1} in bulk solution.[20] The lowest pH-independent rate constant for intramolecular cyclization of the αHL-DTT adduct (k_3) in our experiments was 174 s^{-1} for the αHL-DTT thiolate at position 113. We estimated the minimum effective concentration of the αHL-DTT thiolate to be 4.7 M ≈ (174 s^{-1}) / (37 M^{-1} s^{-1}), which is ~47 times greater than the concentration of a single freely diffusing molecule within the β barrel of αHL (~100 mM).[21] Because the αHL-DTT thiolates and a free DTT molecule have similar pK_a values, their statistical probabilities of deprotonation should be similar. As a result, intramolecular cyclization of the αHL-DTT adduct should be the dominant reaction pathway over the intermolecular attack of DTT onto a αHL-DTT adduct.

Effective molarity is defined as k_{intra}/k_{inter}, where k_{inter} and k_{intra} are the rate constants of analogous inter- and intramolecular reactions. Effective molarity measures the ease of intramolecular cyclization, corrected for the inherent reactivity of the end groups.[22] The effective molarity for the cyclization of bifunctional 6-membered rings is approximately 20 M.[22] Discrepancies within an order of magnitude are not uncommon when comparing reactions in bulk solution and those within the αHL nanoreactor, and may reflect small perturbations in kinetics.[21] For example, with the DTT adduct at a cysteine foothold, steric hindrance owing to proximity to the protein wall may be encountered. Hence, rate constants k_3 obtained from our single-molecule analyses correlate reasonably well with reactions conducted in bulk solution.

7. Biopolymer translocation in $(E111Q\text{-}2C)_1WT_6$

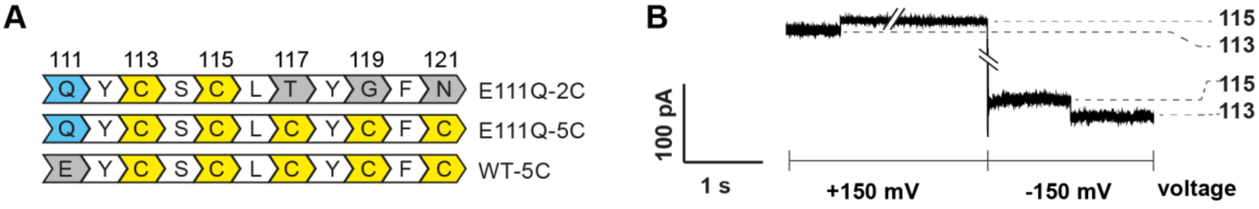

C

	(pH 9.5) $(E111Q\text{-}2C)_1WT_6$ k^f (s^{-1})	(pH 8.5) $(E111Q\text{-}2C)_1WT_6$ k^f (s^{-1})	(pH 8.5) $(WT\text{-}5C)_1WT_6$ k^f (s^{-1})	$k_{E111Q,pH\ 9.5}$ / $k_{E111Q,pH\ 8.5}$	$k_{E111Q,pH\ 8.5}$ / $k_{WT,pH\ 8.5}$
113-to-115 (+150 mV)	1.4 ± 0.2	0.34 ± 0.07	0.044 ± 0.009	4.2	7.7
115-to-113 (-150 mV)	0.11 ± 0.003	0.040 ± 0.010	0.054 ± 0.010	2.8	0.7

Figure S8. Hopping rate acceleration in the $(E111Q\text{-}2C)_1WT_6$ track. (A) Comparison of the primary sequences of the protein tracks investigated. (B) On the two-cysteine track, a single hopping step was observed for both +150 mV and -150 mV. Conditions: 2M KCl, 50 mM AMPSO, pH 9.5, 20 µM EDTA, 20 ± 1 °C. (C) Hopping rates were accelerated on increasing the pH from 8.5 to 9.5. Under +150 mV, the 113-to-115 hopping rate was increased on introduction of the E111Q mutation. Under -150 mV, the 115-to-113 hopping rate remained unchanged.

The $(E111Q\text{-}2C)_1WT_6$ nanoreactor carrying the engineered two-cysteine track E111Q-M113C-T115C was assembled. Translocation of a molecular hopper carrying a multi-adenosine 40-mer cargo was investigated in the two-cysteine track.

The E111Q mutation was observed to promote faster 113-to-115 stepping under +150 mV ($k^f_{113\text{-}115}$ = 0.34 ± 0.07 s^{-1} (E111Q-2C), 0.044 ± 0.010 s^{-1} (WT-5C)), while 115-to-113 stepping under -150 mV remained slow ($k^f_{115\text{-}113}$ = 0.040 ± 0.010 s^{-1} (E111Q-2C), 0.054 ± 0.010 s^{-1} (WT-5C)). Increasing the pH produced faster 113-to-115 and 115-to-113 stepping under +150 mV and -150 mV respectively ($k^f_{113\text{-}115}$ = 1.4 ± 0.2 s^{-1} (pH 9.5), 0.34 ± 0.07 s^{-1} (pH 8.5) and $k^f_{115\text{-}113}$ = 0.11 ± 0.003 s^{-1} (pH 9.5), 0.04 ± 0.01 s^{-1} (pH 8.5)). These results are consistent with the rate enhancements observed in the engineered five-cysteine track in the $(E111Q\text{-}5C)_1WT_6$ nanoreactor.

Experimental Details

General

All reagents were purchased from Sigma-Aldrich unless stated otherwise. 1,2-Diphytanoyl-sn-glycerol-3-phosphocholine (DPhPC) was purchased from Avanti Polar Lipids. OPSSG was synthesized as described previously.[23]

Plasmid preparation

The construction of single-cysteine plasmids pT7-αHL-113C-D8H6 (M113C), pT7-αHL-115C-D8H6 (T115C), pT7-αHL-117C-D8H6 (T117C), pT7-αHL-119C-D8H6 (G119C), pT7-αHL-121C-D8H6 (N121C), pT7-αHL-123C-D8H6 (N123C), which encode the single cysteine mutant subunits in the αHL heteroheptamers, have previously been reported.[19] The construction of five-cysteine plasmid pT7–αHL–113C115C117C119C121C-D8H6 (M113C-T115C-T117C-G119C-N121C) has also been previously reported.[19,24]

Plasmids encoding mutants bearing the E111Q and E111S mutation were prepared by site-directed mutagenesis (Agilent QuikChange II XL Site-Directed Mutagenesis Kit) by a modification of the procedure suggested in the kit. Each PCR (25 µL) was set up by mixing the following reagents in the order listed: 10× reaction buffer (2.5 µL), nuclease-free water (to make up the final reaction volume to 25 µL), double-stranded plasmid DNA template (5 µL, 5 ng, 1 ng/µL), mutagenic primers (62.5 ng of each primer), dNTP mix (0.5 µL), QuikSolution (1.5 µL) and PfuUltra HF DNA polymerase (0.5 µL, 2.5 U/µL). PCRs were carried out with the following program: 95 °C for 5 min, 18 cycles of 95 °C (50 s), 55 °C (E111Q-M113C and E111S-M113C) or 58 °C (E111Q-M113C-T115C and E111Q-M113C-T115C-T117C-G119C-N121C) (50 s for all mutants), 68 °C (5 min), followed by a final extension at 68 °C for 7 min. The PCRs were then cooled on ice for 2 min and then the template DNA was digested with DpnI (0.5 µL, supplied with the kit) at 37 °C for 1 h. Plasmids containing the mutant genes were generated by transforming *E. coli* XL-10 Gold ultracompetent cells with the PCR product. DNA sequences of the genes were verified (Source BioScience).

The mutants, primers and DNA templates in each site-directed mutagenesis reaction were as follows: E111Q-M113C (DNA template: M113C; forward primer: 5’ CGATTGATACAAAA**CAG**TATTGCAGTACGTTAACGTACGGATTCAACGG 3’; reverse primer: 5’ CCGTTGAATCCGTACGTTAACGTACTGCAATA**CTG**TTTTGTATCAATCG 3’), E111S-M113C (DNA template: M113C; forward primer: 5’ CGATTGATACAAAA**TCG**TATTGCAGTACGTTAACGTACGGATTCAACGG 3’; reverse primer: 5’ CCGTTGAATCCGTACGTTAACGTACTGCAATA**CGA**TTTTGTATCAATCG 3’), E111Q-2C (DNA template: M113C-T115C; forward primer: 5’ CGATTGATACAAAA**CAG**TATTGCAGTTGCTTAACGTACGGATTCAACGG 3’; reverse primer: 5’ CCGTTGAATCCGTACGTTAAGCAACTGCAATA**CTG**TTTTGTATCAATCG 3’), E111Q-5C (DNA template: M113C-T115C-T117C-G119C-N121C; forward primer: 5’ CGATTGATACAAAA**CAG**TATTGTAGTTGCTTATGCTATTGCTTCTGCGG 3’; reverse primer: 5’ CCGCAGAAGCAATAGCATAAGCAACTACAATA**CTG**TTTTGTATCAATCG 3’). Bolded codons represent the target sites for mutagenesis.

Protein preparation

The heptameric *S. aureus* αHL pores were prepared by a modification of the method previously described.[25] Heteroheptamers $(M113C)_1WT_6$, $(T115C)_1WT_6$, $(T117C)_1WT_6$, $(G119C)_1WT_6$, $(N121C)_1WT_6$, $(N123C)_1WT_6$, $(E111Q\text{-}M113C)_1WT_6$, $(E111S\text{-}M113C)_1WT_6$, $(WT\text{-}5C)_1WT_6$, $(E111Q\text{-}2C)_1WT_6$ and $(E111Q\text{-}5C)_1WT_6$ were prepared according to the procedures below.

***In vitro* transcription and translation and preparation of αHL mutants**

αHL monomers were prepared by the *E. coli in vitro* transcription and translation (IVTT) system (*E. coli* T7 S30 Extract System for Circular DNA, Cat #L1130, Promega). Prior to use, the T7 S30 extract provided in the kit was treated with 1 µL rifampicin (1 µg mL^{-1}, final concentration) to suppress transcription by *E. coli* RNA polymerase. A standard reaction comprised: DNA plasmid mixture (<4 µg, DNA plasmids in a 7:1 ratio of background: mutant), amino acid mixture without methionine (5 µL, as supplied in the kit), S30 premix without amino acids (20 µL, as supplied in the kit), [^{35}S]methionine (2 µL) and T7 S30 extract, circular (15 µL, as supplied in the kit). The reaction mixture was supplemented with nuclease-free water to bring the final volume to 50 µL. The mixture was incubated at 37°C for 1 h.

After the incubation period, rabbit red blood cell membranes (rRBCm) (2 µL, ~ 1 mg protein /mL) were added to the reaction mixture to induce heptamerization of the αHL monomers. The mixture was then incubated at 37°C for another 1 h.

After the second incubation period, MBSA buffer (1 mL; 3-morpholinopropane-1-sulfonic acid (10 mM), NaCl (150 mM), bovine serum albumin (1 mg/mL), pH 7.4) was added to the reaction mixture. The mixture was centrifuged at 12000 RCF for 10 min at 4°C. The supernatant was removed, and the pellet solubilized at room temperature with 2× Laemmli sample buffer (25 µL) which had been supplemented with 2-mercaptoethanol. Before loading the samples, a 5.5 % SDS/PAGE gel was pre-run with 1× Tris-Glycine SDS (TGS) running buffer containing DTT (2 mM) and sodium thioglycolate (1 mM). The resuspended pellets were then loaded onto the gel and electrophoresed at 70 V overnight (13 h).

αHL heptamers composed of different numbers of mutant subunits were separated in the gel based on their different electrophoretic mobilities which were determined by the number of octa-aspartate (D8) tails. Hence, the top band corresponded to homoheptamers WT_7, the second band corresponded to $(mutant)_1WT_6$ and so on, with consecutive bands having a WT subunit replaced with a mutant subunit. In this work, the second band from the top contained the desired protein pore.

To extract the protein pores, the gel was first dried under vacuum onto Whatman 3MM filter paper for 5 h at 50 °C. The dried gel was then exposed to photographic film (Kodak Bio Max MR autoradiography film) for 8 h and the developed film was used to locate the target protein bands in the gel. The desired protein bands were excised and rehydrated in TE buffer (300 µL; Tris·HCl (10 mM), ethylenediaminetetraacetic acid (EDTA) (1 mM), pH 8.0) for 1 h at room temperature. The paper was then removed, and the gel crushed with a plastic pestle. The resulting suspension was filtered through a 0.2 µm hydrophilic membrane filter (Proteus Mini Clarification Spin Column, Generon). The filtrate was stored in 10 µL aliquots at -80 °C.

Single-channel electrical recordings

Single-channel recordings were carried out in a planar bilayer apparatus as previously described.[26] A single αHL pore was allowed to insert into the bilayer. DTNB or OPSSG was introduced from the *cis* compartment, and DTT from the *trans* compartment. Pre-weighed samples of solid DTT and DTNB were prepared and stored at -20°C. Before an experiment, buffer was added to generate a 500 mM DTT stock and a 20 mM DTNB stock, which were kept on ice. The experiments were conducted at different pH values: pH 8.0, 8.4 and 8.5 (2 M KCl, 100 mM HEPBS, 20 μM EDTA), and 8.8, 9.0 and 9.5 (2 M KCl, 50 mM AMPSO, 20 μM EDTA). All reactions were carried out with $[DTNB]_{cis}$ = $[DTT]_{trans}$ = 50 μM, with the exception of Cys-113 where $[DTNB]_{cis}$ = $[DTT]_{trans}$ = 200 μM, and Cys-123 where $[DTNB]_{cis}$ = 100 μM and $[DTT]_{trans}$ = 50 μM.

Ionic currents were recorded by using a patch clamp amplifier (Axopatch 200B, Axon Instruments), and filtered with a low-pass Bessel filter (80 dB/decade) with a corner frequency of 10 kHz. Signals were digitized with a Digidata 1320A digitizer (Molecular Devices) at an acquisition frequency of 50 kHz. The current traces were processed with Clampfit 10.7 (Molecular Devices). Current traces were idealized by using Clampfit 10.3 (Molecular Devices). The idealized data were analyzed with QuB 2.0 software (www.qub.buffalo.edu).[27] Dwell time analysis and rate constant determinations were performed by using the maximum interval likelihood (MIL) algorithm of QuB.[18]

References

[1] P. J. Bond, A. T. Guy, A. J. Heron, H. Bayley, S. Khalid, *Biochemistry* **2011**, *50*, 3777–3783.
[2] J. Wang, W. Wang, P. A. Kollman, D. A. Case, *J. Mol. Graph. Model.* **2006**, *25*, 247–260.
[3] J. Wang, R. M. Wolf, J. W. Caldwell, P. A. Kollman, D. A. Case, *J. Comput. Chem.* **2004**, *25*, 1157–1174.
[4] M. J. Frisch, G. W. Trucks, H. B. Schlegel, G. E. Scuseria, M. a. Robb, J. R. Cheeseman, G. Scalmani, V. Barone, G. a. Petersson, H. Nakatsuji, X. Li, M. Caricato, a. V. Marenich, J. Bloino, B. G. Janesko, R. Gomperts, B. Mennucci, H. P. Hratchian, J. V. Ortiz, a. F. Izmaylov, J. L. Sonnenberg, Williams, F. Ding, F. Lipparini, F. Egidi, J. Goings, B. Peng, A. Petrone, T. Henderson, D. Ranasinghe, V. G. Zakrzewski, J. Gao, N. Rega, G. Zheng, W. Liang, M. Hada, M. Ehara, K. Toyota, R. Fukuda, J. Hasegawa, M. Ishida, T. Nakajima, Y. Honda, O. Kitao, H. Nakai, T. Vreven, K. Throssell, J. a. Montgomery Jr., J. E. Peralta, F. Ogliaro, M. J. Bearpark, J. J. Heyd, E. N. Brothers, K. N. Kudin, V. N. Staroverov, T. a. Keith, R. Kobayashi, J. Normand, K. Raghavachari, a. P. Rendell, J. C. Burant, S. S. Iyengar, J. Tomasi, M. Cossi, J. M. Millam, M. Klene, C. Adamo, R. Cammi, J. W. Ochterski, R. L. Martin, K. Morokuma, O. Farkas, J. B. Foresman, D. J. Fox, **2016**, Gaussian 16, Revision C.01, Gaussian, Inc., Wallin.
[5] M. J. Abraham, T. Murtola, R. Schulz, S. Páll, J. C. Smith, B. Hess, E. Lindah, *SoftwareX* **2015**, *1–2*, 19–25.
[6] K. Lindorff-Larsen, S. Piana, K. Palmo, P. Maragakis, J. L. Klepeis, R. O. Dror, D. E. Shaw, *Proteins Struct. Funct. Bioinforma.* **2010**, *78*, 1950–1958.
[7] D. J. Price, C. L. Brooks, *J. Chem. Phys.* **2004**, *121*, 10096–10103.
[8] G. Bussi, D. Donadio, M. Parrinello, *J. Chem. Phys.* **2007**, *126*, DOI 10.1063/1.2408420.
[9] M. Parrinello, A. Rahman, *J. Appl. Phys.* **1981**, *52*, 7182–7190.
[10] T. Darden, D. York, L. Pedersen, *J. Chem. Phys.* **1998**, *98*, 10089.
[11] U. Essmann, L. Perera, M. L. Berkowitz, T. Darden, H. Lee, L. G. Pedersen, *J. Chem. Phys.* **1995**, *103*, 8577–8593.
[12] B. Hess, H. Bekker, H. J. C. Berendsen, J. G. E. M. Fraaije, *J. Comput. Chem.* **1997**, *18*, 1463–1472.
[13] O. S. Smart, J. M. Goodfellow, B. A. Wallace, *Biophys. J.* **1993**, *65*, 2455–2460.
[14] O. S. Smart, J. G. Neduvelil, X. Wang, B. A. Wallace, M. S. P. Sansom, *J. Mol. Graph.* **1996**, *14*, 354–360.
[15] R. Singh, G. M. Whitesides, in *Sulphur-Containing Funct. Groups*, John Wiley & Sons, Inc., Chichester, UK, **1993**, pp. 633–658.
[16] P. W. Riddles, R. L. Blakeley, B. Zerner, *Anal. Biochem.* **1979**, *94*, 75–81.
[17] E. F. V. Scriven, R. Murugan, in *Kirk-Othmer Encycl. Chem. Technol.*, John Wiley & Sons, Inc., Hoboken, NJ, USA, **2005**.
[18] F. Qin, A. Auerbach, F. Sachs, *Biophys. J.* **1996**, *70*, 264–280.
[19] G. S. Pulcu, E. Mikhailova, L.-S. Choi, H. Bayley, *Nat. Nanotechnol.* **2015**, *10*, 76–83.
[20] R. P. Szajewski, G. M. Whitesides, *J. Am. Chem. Soc.* **1980**, *102*, 2011–2026.
[21] H. Bayley, T. Luchian, S.-H. Shin, M. B. Steffensen, in *Single Mol. Nanotechnol.* (Eds.: R. Rigler, H. Vogel), Springer Berlin Heidelberg, **2008**, pp. 251–277.
[22] C. Galli, L. Mandolini, *European J. Org. Chem.* **2000**, *2000*, 3117–3125.
[23] Z. Yuan, Y. Zheng, B. Yu, S. Wang, X. Yang, B. Wang, *Org. Lett.* **2018**, *20*, 6364–6367.
[24] Y. Qing, S. A. Ionescu, G. S. Pulcu, H. Bayley, *Science* **2018**, *361*, 908–912.
[25] O. Braha, B. Walker, S. Cheley, J. J. Kasianowicz, L. Song, J. E. Gouaux, H. Bayley, *Chem. Biol.* **1997**, *4*, 497–505.
[26] M. Montal, P. Mueller, *Proc. Natl. Acad. Sci. U. S. A.* **1972**, *69*, 3561–3566.
[27] C. Nicolai, F. Sachs, *Biophys. Rev. Lett.* **2013**, *08*, 191–211.

Author Contributions

Z. Bo, Z.H. Lim, F. Duarte, H. Bayley, and Y. Qing conceived the project. Z. Bo, Z.H. Lim and Y. Qing carried out the experiments and data analysis. Z. Bo, and F. Duarte conducted the computational simulations. Z. Bo, Z.H. Lim, F. Duarte, H. Bayley, and Y. Qing wrote the paper.
